# Improved prime editing allows for routine predictable gene editing in *Physcomitrium patens*

**DOI:** 10.1093/jxb/erad189

**Published:** 2023-05-27

**Authors:** Pierre-François Perroud, Anouchka Guyon-Debast, Josep M Casacuberta, Wyatt Paul, Jean-Philippe Pichon, David Comeau, Fabien Nogué

**Affiliations:** Université Paris-Saclay, INRAE, AgroParisTech, Institut Jean-Pierre Bourgin (IJPB), 78000 Versailles, France; Université Paris-Saclay, INRAE, AgroParisTech, Institut Jean-Pierre Bourgin (IJPB), 78000 Versailles, France; Centre for Research in Agricultural Genomics CSIC-IRTA-UAB-UB, Campus UAB, Edifici CRAG, Bellaterra, 08193 Barcelona, Spain; Limagrain Europe, Centre de Recherche de Chappes, 63720 Chappes, France; Limagrain Europe, Centre de Recherche de Chappes, 63720 Chappes, France; Limagrain Europe, Centre de Recherche de Chappes, 63720 Chappes, France; Université Paris-Saclay, INRAE, AgroParisTech, Institut Jean-Pierre Bourgin (IJPB), 78000 Versailles, France; Cardiff University, UK

**Keywords:** EpegRNA, genome editing, *Physcomitrium patens*, prime editing, pseudoknot, split prime editing

## Abstract

Efficient and precise gene editing is the gold standard of any reverse genetic study. The recently developed prime editing approach, a modified CRISPR/Cas9 [clustered regularly interspaced palindromic repeats (CRISPR)/CRISPR-associated protein] editing method, has reached the precision goal but its editing rate can be improved. We present an improved methodology that allows for routine prime editing in the model plant *Physcomitrium patens*, whilst exploring potential new prime editing improvements. Using a standardized protoplast transfection procedure, multiple prime editing guide RNA (pegRNA) structural and prime editor variants were evaluated targeting the *APT* reporter gene through direct plant selection. Together, enhancements of expression of the prime editor, modifications of the 3ʹ extension of the pegRNA, and the addition of synonymous mutation in the reverse transcriptase template sequence of the pegRNA dramatically improve the editing rate without affecting the quality of the edits. Furthermore, we show that prime editing is amenable to edit a gene of interest through indirect selection, as demonstrated by the generation of a *Ppdek1*^*0*^ mutant. Additionally, we determine that a plant retrotransposon reverse transcriptase enables prime editing. Finally, we show for the first time the possibility of performing prime editing with two independently coded peptides.

## Teaser

Using a strong expression system and engineered guide RNAs, we present two routine genome editing approaches in the moss *Physcomitrium patens*: prime editing and the split prime editing.

## Introduction

Prime editing (PE) is a CRISPR/Cas9- [clustered regularly interspaced palindromic repeats (CRISPR)/CRISPR-associated protein] based DNA editing approach that allows for single base or multiple base changes and small deletions and insertions at a defined locus (Anzalone *et al.*, 2019). The PE approach is based on the use of a modified Cas9 enzyme, the prime editor, which contains two effector domains, the *Streptococcus pyogenes* (H840A) nickase Cas9 (nCas9) and an engineered reverse transcriptase (e.g. the pentamutant D200N/L603W/T330P/T306K/W313F of the moloney murine leukemia virus reverse transcriptase referred to as RT-_MMLV_ hereafter). The nCas9 conserves the DNA search ability of the Cas9 but generates only a single strand DNA break at the target site, reducing the mutagenic risk of the presence of this protein in the cell. RT-_MMLV_ allows the reverse transcription of the desired edit to be inserted at the target site. The guide RNA, renamed prime editing guide RNA (pegRNA), is modified to contain at its 3ʹ end the template of the desired edit (RT template) and a short sequence, immediately adjacent to the editing site, aiming to anchor the RT template at the locus to be edited (primer-binding site or PBS) (Anzalone *et al.*, 2019). Initially developed in mammalian cells, this approach has been extended to other kingdoms, including the green lineage. Cereals, especially rice, have played and still play a driving role in PE development in plants, with the first of multiple reports in 2020 (Lin *et al.*, 2020). PE appears to be functional in all the plants in which it has been attempted (Molla *et al.*, 2021), and the list of the plants successfully submitted to the PE procedure steadily increases (see the most recent in several legumes species, Biswas *et al.*, 2022).

Recently, PE has been shown to function in *Physcomitrium patens* in the same way as in other plants (Perroud *et al.*, 2022). Although faithful at its edited locus and generating fewer unwanted off-target events than the standard CRISPR/Cas9 approach (G. Liu *et al*., 2022), the use of PE has been hindered initially by a low overall efficiency in animal and plant cells alike, prompting the exploration of several avenues to improve its editing rate (for a recent review, on the topic in the plant context, see Ahmad *et al.*, 2023). The RNA integrity in prokaryote and eukaryote cells is the constant target of different types of nuclease whether this is for endogenous functions, to control the transcription and/or translation regulation, or in answer to exogenous threats such as by a phage and virus (Tatosyan *et al.*, 2020). Hence, in the context of the PE biotechnology application, the protection of the pegRNA, which contains the editing matrix at its 3ʹ end, has been identified as an opportunity to improve PE efficiency (Nelson *et al.*, 2022). The 3ʹ extension of pegRNA with a short RNA sequence able to form a pseudoknot structure was shown to improve PE efficiency in mammalian systems (Chen *et al.*, 2021; Nelson *et al.*, 2022) and in rice (X. Li *et al*., 2022b; Zong *et al.*, 2022). The exact mechanism behind this protection remains to be deciphered, but in prokaryotes the presence of pseudoknots in RNA sequences hinders the RNA scanning mechanism of RNase H, increasing the RNA stability significantly (Richards and Belasco, 2021), an indication that pseudoknot topology can act directly on the RNase H endonuclease activity. Alternatively, secondary RNA structures like the pseudoknot can bind a protein or a protein complex that in turn could act as a protectant against nuclease activity (Akiyama *et al.*, 2016). RNA pseudoknot structures are naturally found in the RNA of certain groups of RNA viruses (Brierley *et al.*, 2007) and play, for example, a key role in transcriptional slippage in the *Potyviridae*, a major group of plant viruses (Olspert *et al.*, 2015). The turnip yellow mosaic virus (TYMV) RNA 3ʹ end forms a tRNA-like (tls) structure (Rietveld *et al.*, 1982) that plays an important role in virus replication and virus spread through its host. The role of this structure appears multifunctional and is not fully elucidated, but its last 37 nucleotides form a pseudoknot structure (Colussi *et al.*, 2014) akin to those used by Nelson *et al.* (2022), and thus could potentially be used to stabilize pegRNA.

The addition of extraneous silent mutation in the RT template is another type of pegRNA modification that has shown promise in improving PE rates in mammalian cells. Increasing the mismatch between the edited template and the wild-type sequence appears to favor the edited DNA strand insertion at the site of the single strand DNA break by evasion of the mismatch repair mechanism (MMR) (Chen *et al.*, 2021). This explanation has been further reinforced by the observation of an increase in the PE rate in the absence of active MMR in mammalian cells (Ferreira da Silva *et al.*, 2022). This approach also improved the PE rate in rice, but the rate improvement appeared to be pegRNA specific (Xu *et al.*, 2022).

The improvement of the prime editor polypeptide has also been investigated (Chen and Liu, 2023). For example, Xu and colleagues showed that a prime editor with an inverted RT_-MMLV_–nCas9 topological order was functional in rice, and this modification, in combination with additional silent mutation in the RT template, could outperform the standard nCas9–RT_-MMLV_ (Xu *et al.*, 2022). More pertinent to the present study, in plants, two alternative RTs have been tested in a PE context in rice, the *Escherichia coli* BL21 retron RT and the cauliflower mosaic virus RT. Both were functional in PE, but displayed significantly lower rates of editing as compared with RT-_MMLV_ (Lin *et al.*, 2020). More surprising, PE was successfully achieved by independently expressing the prime editor as two different peptides in mammalian cells (B. Liu *et al*., 2022; Feng *et al*., 2023; Grünewald *et al*., 2023); indicating that the physical link between the two functional domains of the prime editor is not required to generate a specific PE. The modular nature of this approach, referred to as split PE (sPE), will ease the rapid test of prime editor variant domains (Grünewald *et al*., 2023).

In the present study we show that the addition of different 3ʹ extensions at the end of pegRNA boosts the PE rate in the moss *P. patens* without affecting the fidelity of the editing. Moreover, this increase is high enough to allow for indirect selection of an edit, as demonstrated through the PE-generated *dek*^*0*^ mutant, opening up the PE approach for practical efficient and clean editing of virtually any locus in the genome of *P. patens*. We show that combining the addition of silent mutations in the RT template with the pegRNA 3ʹ extension can improve PE efficiency further. We also report that the native RT from an endogenous plant retrotransposon *Tnt1* displays PE activity in association with nCas9. Finally, we report the first successful gene editing using sPE in a plant, paving the way to a systematic domain-oriented analysis of the prime editors.

## Materials and methods

### Plant material and culture

We used the *P. patens* ecotype Gransden pedigree Versailles (Haas *et al.*, 2020) and the mutant *P. patens dek*^*0*^ (Johansen *et al.*, 2016) in this study. Tissue was routinely maintained and propagated on PpNH_4_ medium either by tissue picking or through tissue blending in sterile purified water (Schaefer *et al.*, 1991). Culture chamber conditions were set at 60% humidity, temperature at 22 °C with a long-day light cycle of 16 h of light (quantum irradiance of 80 μmol m^−2^ s^−1^) and 8 h of dark. Spot inoculum phenotypic analyses were performed on PpNO_3_ medium, with replicated wild-type and mutants growing in co-culture on the same plate and triplicated plate replication. Plant imaging was performed after 14 d of growth.

### Vector design and assembly

**Fig. 1.**
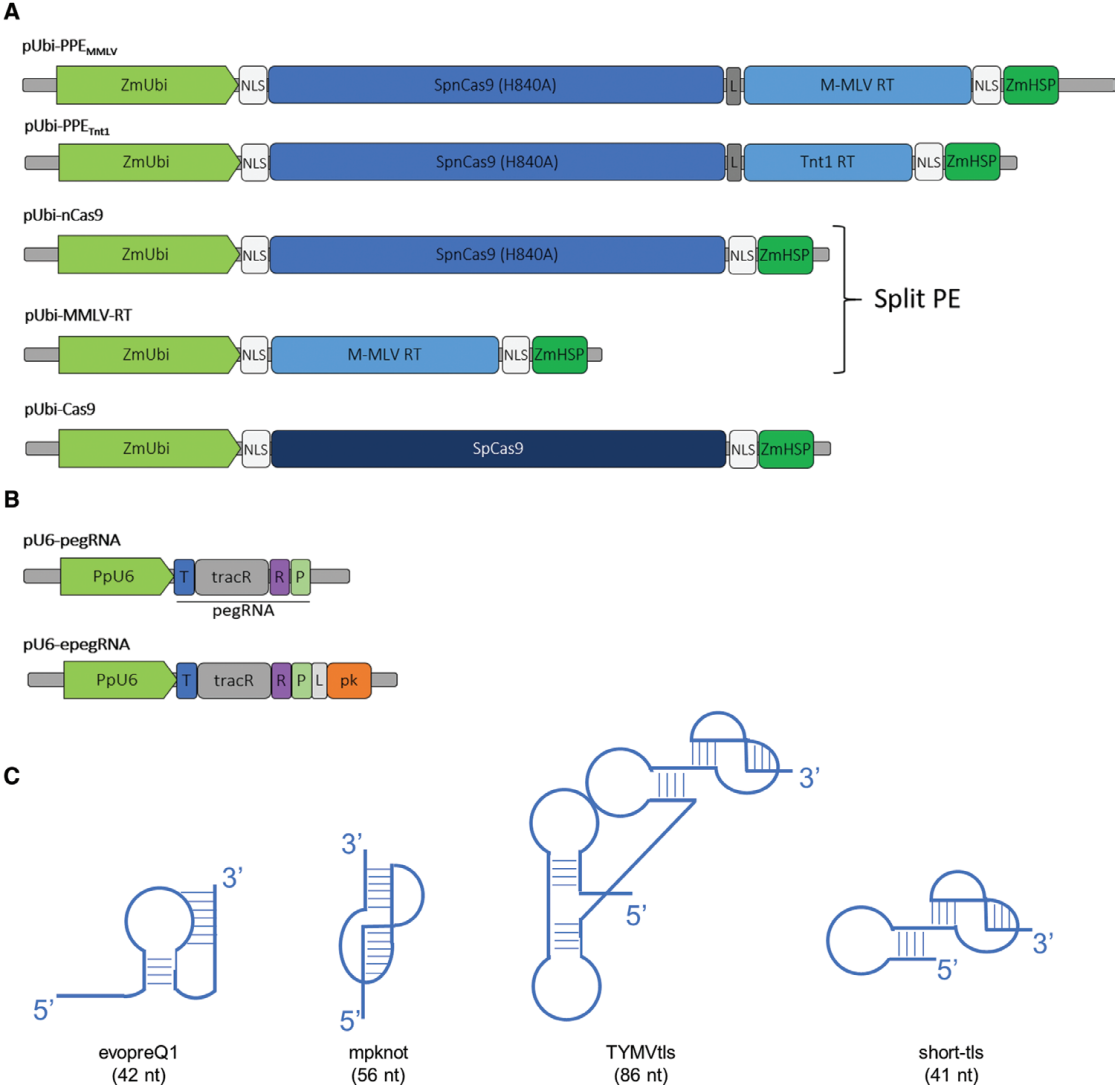
Graphic representation of the tools used for the prime editing strategy. (A) Schematic representation of the plant prime editors (PPEs) used for transfection of moss protoplasts: pUbi-PPEMMLV in fused or split version and pUbi-PPETnt1. The active Cas9 (pUbi-Cas9) was used as control. (B) Schematic representation of pegRNA constructs used for transfection of moss protoplasts: pU6-pegRNA or pU6-epegRNA constructs. T, target sequence; tracR, tracRNA (scaffold); R, RT template; P, primer-binding site (PBS); L, linker; pk, pseudoknot motif. (C) Schematic representation of secondary structure of RNA extension added at the 3ʹ end of the pegRNA. Four alternative pseudoknot motifs were used: evopreQ1 (42 nt), mpknot (56 nt), TYMV-tls (86 nt), and short-tls (41 nt). The size of boxes is not to scale.

The plant sequence reference is based on the Phytozome13 dataset at https://phytozome-next.jgi.doe.gov/ (Goodstein *et al.*, 2012). Constructs used in this study were either completely *de novo* synthesized or assembled from synthesized domains cloned in a commercial vector (GenScript, Piscataway, NJ, USA). In all enzyme-expressing constructs, transcripts were controlled by the maize ubiquitin promoter (Christensen and Quail, 1996), a strong promoter in *P. patens*, and the *Zm*HSP terminator. For all the editing enzymes, the transcript-coding sequence was optimized using a monocotyledon coding bias. The three different PPE (plant prime editing) enzyme configurations (see Fig. 1A and Supplementary Fig. S1 for full vector maps) used in this study are similar to the protein used by Anzalone *et al.* (2019). They are composed of two functional domains, the nCas9 (H840A) domain and the RT domain. pUbi-PPE expresses the fusion protein comprising nCas9 and the M-MLV RT (D200N, T306K, W313F, T330P, L603W) according to Anzalone *et al.* (2019). pUbi-PPE_tnt_ expresses the fusion protein comprising the nCas9 and the RT domain of the *Nicotiana tabacum* transposable element *Tnt1* (Grandbastien *et al.*, 1989) (see Supplementary Fig. S2 for the full RT_tnt-1_ sequence). pUbi-nCas9 expresses the nCas9 domain and pUbi-MMLV-RT expresses the M-MLV RT (D200N, T306K, W313F, T330P, L603W). The control vector pUbi-Cas9 expresses the canonical Cas9. Finally, two nuclear localization signal (NLS)-coding sequences (either SV40 or nucleoplasmin type) were added to the N-terminus and C-terminus, respectively, of every polypeptide to ensure proper nuclear targeting of the enzymes.

The pegRNAs, designed to target and edit the *PpAPT* gene (Pp3c8_16590) and the *PpDEK1* gene (Pp3c17_17550), were designed with the previously used expression structure (Perroud *et al.*, 2022) (see Fig. 1B for schematic vector representation, Supplementary Fig. S3 for specific editing details, and Supplementary Table S1 for specific target and RT template sequences). The transcription of all pegRNAs and epegRNAs (for engineered pegRNAs) was carried out using the *Pp*U6 promoter (Collonnier *et al.*, 2017), and transcript termination was assured by the SUP4 terminator (Chédin *et al.*, 1998). PegRNA expression constructs contained a common single guide RNA (sgRNA) scaffold (see Supplementary Fig. S4A pegAPT#3 for a representative pegRNA-expressing vector map). Compared with standard pegRNAs, epegRNAs differ only by the presence of a 3ʹ extension aimed to improve the guide efficiency (see Fig. 1C for a schematic representation, Supplementary Table S2 for sequence information, and Supplementary Fig. S4B epegAPT#3-evopreQ1 for a representative epegRNA-expressing vector map). The four 3ʹ extensions tested are formed by a small adaptor sequence (8 nt) and the stable RNA pseudoknot-forming RNA sequence as used by Nelson and collaborators (Chen *et al.*, 2021). The 3ʹ extension evopreQ1 and mpknot sequences are identical to those used by Nelson *et al.* (2022). The third 3ʹ extension tested is referred to as TYMVtls. This pseudoknot-generating RNA sequence is naturally found at the 3ʹ end of TYMV (Colussi *et al.*, 2014). The fourth 3ʹ extension tested is formed by the last 41 bp of TYMVtls and is named in this study ‘short-tls’. Complete expression units were synthesized (GenScript) and cloned into a pUC57-Amp vector by the manufacturer. Before use, plasmid DNA was ethanol precipitated to ensure sterility for protoplast transfection.

### Moss transfection and selection procedures

Moss protoplast isolation was performed from 6-day-old blended protonemal tissue as previously described (Charlot *et al.*, 2022). Due to the high efficiency of the transfection and editing procedure, protoplast number per transfection was cut by half compared with the previous PE study (Perroud *et al.*, 2022). A total of 180 000 protoplasts were transfected with 7.5–10 µg of circular plasmid DNA per transfection. Equal quantities of each co-transfected plasmid were systematically used regardless of whether the plasmid number was two in the case of direct selection or three in the case of indirect selection (see below for the two selection procedures). After transfection, the dilution of the transfection reaction, its embedding in alginate, and spreading on cellophane disks laid atop of PpNH_4_ medium supplemented with 0.33 M mannitol were performed as previously described (Charlot *et al.*, 2022). Two different selection procedures were used in this study (Supplementary Fig. S5). For direct selection of *apt* mutant plants, cellophane disks with regenerating transfected plants were transferred on PpNH_4_ medium supplemented with 10 μM 2-fluoroadenine (2-FA) (Fluorochem, Hadfield, UK) (Collonnier *et al.*, 2017). Growing plants were counted after 10 d and individually subcultured on fresh PpNH_4_ medium until harvesting for genotyping (Supplementary Fig. S5A). For indirect selection of the *apt* mutant plants and for the selection of the *dek*^*0*^ mutant plants, cellophane disks with regenerating transfected plants were transferred on PpNH_4_ supplemented with 30 mg l^–1^ G418 (Eurobio Scientific, Les Ulis, France) medium. Growing plants were individually isolated after 5–7 d and subcultured on fresh PpNH_4_ medium for 2 weeks before secondary selection, observation, and harvesting for genotyping (Supplementary Fig. S5B).

### PCR and sequence analysis of the edited plants

Supplementary Table S3 lists the primers used in the present study. Moss genomic DNA samples were isolated from 50 mg of fresh tissue in 96-well microtube plates as previously described (Lopez-Obando *et al.*, 2016). Genomic DNA quality was evaluated using primers targeting the *PpRAD51-1* gene, a non-targeted locus, with the primers PpRAD51-1#6 and PpRAD51-1#7. Primers PpAPT#14 and PpAPT#5 were used for the loci targeted by pegAPT#1, #3, and #8, primers PpAPT#60 and PpAPT#61 for the loci targeted by pegAPT#2, and primers PpDEK1-Fwd and PpDEK1-Rev for the locus targeted by pegDEK1. Sequence analysis was performed on PCR fragments by direct Sanger sequencing (Genoscreen, Lille, France) using one of the primers used to amplify the targeted loci.

### Microscopy

Images were acquired with a Zeiss Axio Zoom.V16 Stereo Microscope using the pZen (Blue edition) software. Final panel assembly was performed using Adobe Photoshop.

## Results

### The *Zm*Ubiquitin promoter successfully drives prime editing in *P. patens*

Although the PE approach worked in *P. patens*, the editing rate remained too low to be routinely usable for gene function analysis (Perroud *et al.*, 2022). To improve it, we first tried to improve the prime editor expression by using a strong expression promoter, the *Zm*Ubiquitin promoter. With this aim, we built the construct Ubi-PPE (Fig. 1A; Supplementary Fig. S1A) in which *Zm*Ubiquitin drives a monocotyledon codon-optimized prime editor (Fig. 1A; Supplementary Fig. S1A). The effectiveness of the pUbi-PPE construct PE was evaluated using pegAPT#1, #2, #3, and #8, all pegRNAs (see Supplementary Table S1 for sequence information and Supplementary Fig. S3 for targeting and editing information) previously used to establish PE with a prime editor under the control of the *Os*Act1 promoter (Perroud *et al.*, 2022). These four pegRNAs which target the *PpAPT* gene allow direct positive *apt* mutant selection on 2-FA. After protoplast transfection and direct selection (Supplementary Fig. S5A), the PE mutation rates using pegAPT#1 and #2 (Table 1) were similar to those previously reported with pAct-PPE (Perroud *et al.*, 2022). However, whereas no edited events were obtained with the pAct-PPE promoter using pegAPT#8, pUbi-PPE yielded *apt* mutant plants with similar low yield to pegAPT#1 and #2 (Table 1). With a mutation rate of 0.391% (Table 1), pegAPT#3 also displayed an increased rate between the two prime editor constructs (0.035%, Perroud *et al.* 2022). Additionally, the quality of the edit, for example the achievement of the desired event, remained very high, between 77% and 100% fidelity (Supplementary Fig. S6;Table 1), indicating that this new prime editor vector did not change the quality of PE in *P. patens*. An increase of PE for some pegRNAs with pUbi-PPE compared with pAct-PPE can be attributed to the promoter itself, the codon optimization of the PE, the terminator, or to a combination of the three. Although the advantage conferred by pUbi-PPE in terms of editing rate was not systematic, we used the pUbi-PPE construct as the prime editor reference vector for the remainder of this study.

**Table 1.** Prime editing efficiency and quality of pegRNAs and epegRNAs using the pUbi-PE system in *P. patens*

Name	pUbi-PE^a^		
	2-FA^R^^b^	Mutation frequency (%)^c^	PE % (*n*)^d^
**pegAPT#1**	13	0.007 (±0.001)	77% (13)
**epegAPT#1-evopreQ1**	838	0.578 (±0.072)	76% (45)
**epegAPT#1-mpknot**	1008	0.704 (±0.115)	75% (57)
**epegAPT#1-TYMVtls**	994	0.732 (±0.021)	74% (47)
**pegAPT#2**	17	0.009 (±0.002)	88% (17)
**epegAPT#2-evopreQ1**	112	0.087 (±0.029)	89% (45)
**epegAPT#2-mpknot**	165	0.137 (±0.042)	86% (42)
**epegAPT#2-TYMVtls**	169	0.128 (±0.010)	97% (38)
**pegAPT#3**	423	0.391 (±0.093)	100% (39)
**epegAPT#3-evopreQ1**	2960	2.414 (±0.532)	100% (88)
**epegAPT#3-mpknot**	1804	1.586 (±0.434)	100% (86)
**epegAPT#3-TYMVtls**	1398	1.188 (±0.209)	100% (85)
**pegAPT#8**	12	0.008 (±0.001)	90% (10)
**epegAPT#8-evopreQ1**	90	0.063 (±0.006)	96% (34)
**epegAPT#8-mpknot**	61	0.044 (±0.010)	100% (37)
**epegAPT#8-TYMVtls**	98	0.063 (±0.008)	100% (43)

^a^Data from three independent transfections.

^b^2-FA^R^ stands for the total number of 2-FA-resistant plants obtained during the three transfections.

^c^Mutation frequency is the % of *apt* mutants (2-FA^R^) among the regenerated plants.

^d^Prime editing (PE) efficiency is the % of plants with the expected edits among the *apt* mutants. *n* is the number of sequenced plants.

### The use of an extended pegRNA dramatically increases prime editing efficiency in *P. patens*

**Fig. 2.**
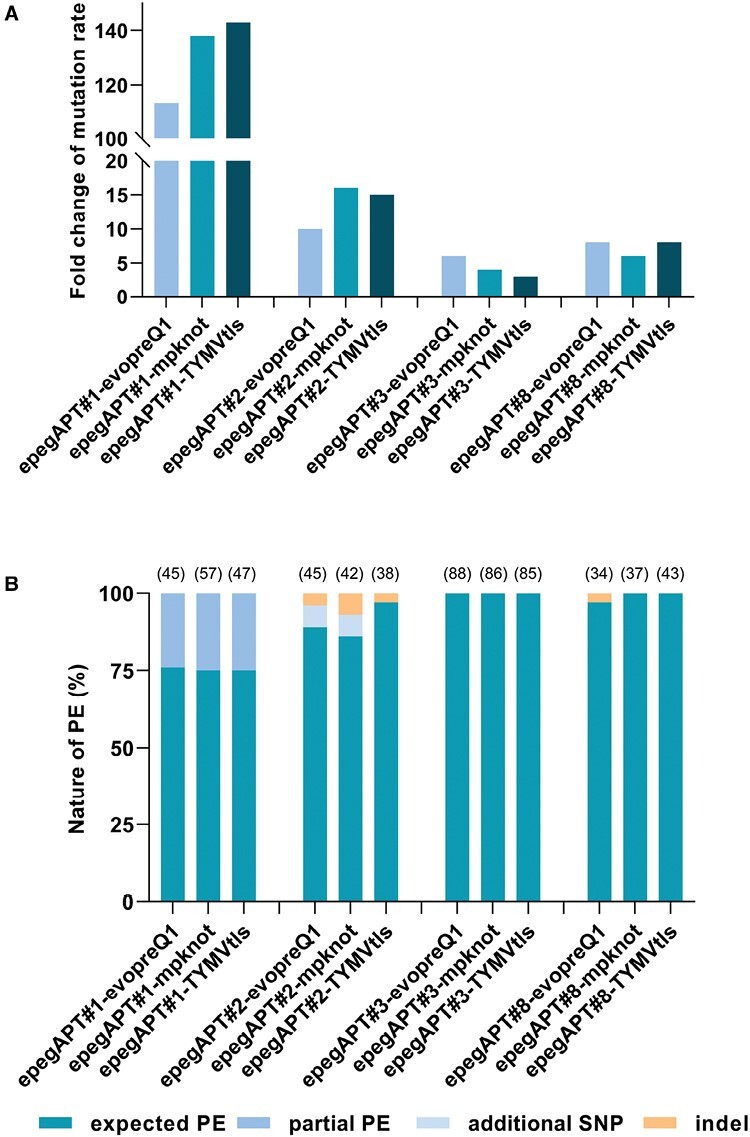
Prime editing (PE) efficiency gain of epegRNA compared with pegRNA and epegRNA PE quality. (A) Fold change of mutation rates were obtained using the three epegRNAs (evopreQ1, mpknot, and TYMVtls) compared with the corresponding pegRNA (three independent experiments). (B) The nature of PE for each epegRNA was established by PCR sequencing of the edited locus (the list of primers is given in Supplementary Table S3; the number of sequenced *apt* mutant plants is indicated in parentheses).

To further increase the PE rate, we evaluated the effect of adding a 3ʹ extension at the end of the pegRNA. We extended the four pegRNAs with three alternative 3ʹ extensions: evopreQ1, which increases the PE rate in mammalian cells (Chen *et al.*, 2021; Nelson *et al.*, 2022) and rice (Zong *et al.*, 2022); mpknot, which increases the PE rate in mammalian cells (Nelson *et al.*, 2022); and TYMVtls, a new potential alternative 3ʹ extension. Such extended pegRNAs are referred to hereafter as epegRNAs in general and with the specific extension as a suffix for each specific guide (e.g. epegAPT#3-evopreQ1). After protoplast transfection and a direct selection procedure (Supplementary Fig. S5), the PE rate increased for all epegRNAs tested regardless of the specific target and the extension type, with a fold change increase varying from 3 to 143 times by comparison with the standard pegRNA (Fig. 2A). The three types of 3ʹ extension conferred increases in PE rate with all tested targets, but the editing rates between targets remained different, as observed with unmodified pegRNAs (Table 1) and in other plants (G. Liu *et al.*, 2022). On the other hand, for the present epegRNA subset, no systematic relationship could be observed in terms of a fold change increased level conferred by the 3ʹ extensions. For example, pegAPT#1, #2, and #8 have a similar low PE rate but the three epegAPT#1s displayed an average 10-fold higher PE rate increase by comparison with epegAPT#2, #3, and #8 (Fig. 2A). Finally, despite the highest PE rate increase observed with epegAPT#1, the highest absolute editing rate remained that of epegAPT#3s, which was already the most efficient pegRNA with the standard pegAPT#3. We subsequently evaluated the quality of the editing at the targeted loci by sequencing between 35 and 86 independent mutants for each target (Fig. 2B; Table 1; Supplementary Fig. S7). The quality of editing with epegRNAs was very high. Interestingly, the epegAPT#3s yielded only perfectly edited plants (Supplementary Fig. S7C), and by-products remained rare with other epegRNAs. The by-products found with the use of the epegAPT#1 were partial editing in which only two (30 cases) or one (one case) of the three desired edits were present (Table 1; Supplementary Fig. S7A). The epegAPT#2 displayed a few aberrant editing events, either small deletions or undesired single nucleotide polymorphisms (SNPs; Table 1; Supplementary Fig. S7B). Notably, the undesired SNP (seven plants, always the same C to G change) was not located in the RT template but before the last base pair of the *trans*-activating CRISPR RNA (tracRNA), indicating the possible base change of a sequence located in this position. Finally, epegAPT#8 displayed three cases of indels. Overall editing quality remained above 74% (Table 1), indicating that the 3ʹ extension of the epegRNA increases the editing rate of the targeted loci without affecting its quality.

Off-target events have been identified as a hazard of CRISPR/Cas9 approaches. Although neither predicted off-targets event (Collonnier *et al.*, 2017; Guyon-Debast *et al.*, 2021; Perroud *et al.*, 2022) nor non-predicted off-target event (Bessoltane *et al.*, 2022) have been detected in *P. patens* with CRISPR/Cas9 approaches so far, we carried out a predicted off-target analysis for the epegAPT#2s to evaluate if peg 3ʹ extension would generate such an event. We PCR-amplified and sequenced nine previously identified potential mistar­geted loci for the epegAPT#2 target sequence (Perroud *et al.*, 2022) in 24 edited plants (eight for each epegRNA#2) and did not detect any off-target event (Supplementary Fig. S8). Thus, the addition of a 3ʹ extension to the pegRNA did not appear to increase the possibility for off-targeting in *P. patens*.

### The high epegRNA primer editing efficiency allows for gene editing through indirect selection

**Fig. 3.**
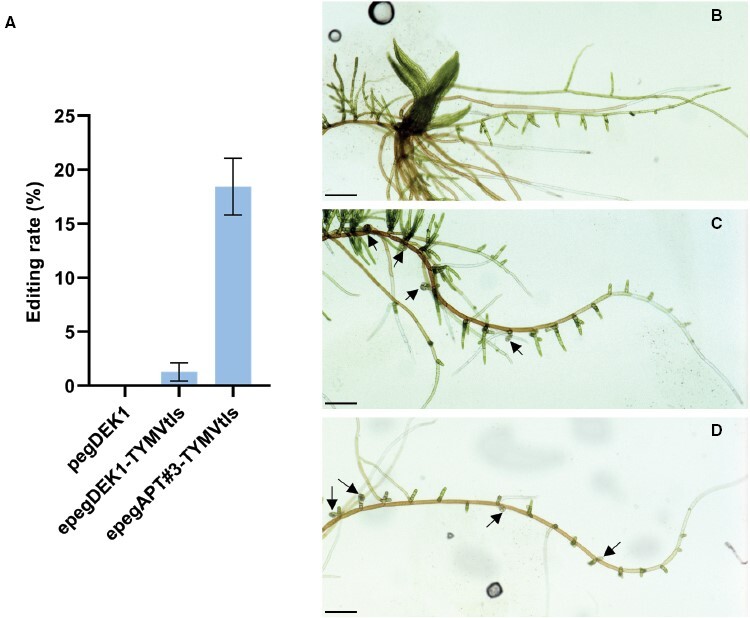
Prime editing approach using indirect selection. (A) Editing rate corresponds to the frequency of mutant plants among the plants resistant to G418 antibiotic (three independent experiments). Error bars=SE. (B–D) Filament from a plant grown from spot inoculum for 18 d on PpNO_3_ medium. (B) Wild-type plant showing a single growing gametophore. (C) *dek*^*0*^ plant obtained by gene targeting displaying multiple aborted buds. (D) *dek*^*0*^ plant obtained by prime editing displaying multiple aborted buds. Arrows: aborting bud. Scale bar: 250 µm.

The high number of edited plants per transfection obtained at the *PpAPT* locus, up to 5% of the regenerated plant total number, prompted us to evaluate if the PE approach could be used to edit loci for which, unlike mutations in the *APT* gene, mutations cannot be directly selected on regenerated protoplasts (Supplementary Fig. S5A). We previously used such a method, ‘indirect selection’, to select knockout or base-edited CRISPR/Cas9-targeted mutants in *P. patens* (Collonnier *et al.*, 2017; Guyon-Debast *et al.*, 2021). With this approach, regenerated transfected protoplasts are selected first with an antibiotic for which a resistance cassette has been co-transfected, and subsequently are evaluated for editing at the targeted locus (Supplementary Fig. S5B). The editing rate is defined in this approach as the number of edited plants among the antibiotic-resistant plants. We tested two different loci in parallel with this approach. The *PpAPT* locus was targeted with epegAPT#3-TYMVtls, an effective epegRNA when using the direct selection approach. To target an independent locus, we performed PE to recapitulate the mutant *dek*^*0*^ obtained previously by a standard homologous recombination approach in *P. patens* (Johansen *et al.*, 2016). *dek*^*0*^ contains two nucleotide changes that convert the PpDEK1 Cys1782 into a serine, leading to a plant without gametophores but overproducing aborting buds, hence easily identifiable visually by comparison with a wild-type plant (Fig. 3B, C). The pegDEK1 and epegDEK1-TYMVtls (see Supplementary Fig. S3 for editing details and Supplementary Table S1 for specific target and RT template sequences) were designed to mediate this change using the PE approach. The protoplast transfection procedure was identical to the direct selection procedure, but required the co-transfection of a plasmid containing a plant neomycin transferase resistance cassette, pBNRF (Schaefer *et al.*, 2010). Upon transfection, regeneration, and selection on a medium containing the antibiotic G418, 100 plants were grown and subsequently assessed for editing either visually in the *dek*^*0*^ trial or with a secondary selection on 2-FA in the *apt* trial. The PE trial using the standard pegDEK1 did not generate any *dek*^*0*^ plants, but both epegAPT#3-TYMVtls and epegDEK1-TYMVtls transfections yielded edited plants (Fig. 3A). On average 18% of antibiotic-selected plants transfected with epegAPT#3-TYMVtls were *apt* mutants and 1.5% of antibiotic-selected plants transfected with epegDEK1-TYMVtls displayed the *dek*^*0*^ mutant phenotype. These were indistinguishable from the original *dek*^*0*^ mutant for protonemal growth and bud formation (Fig. 3A–D; Supplementary Fig. S9). Sanger sequencing of the edited loci showed that regardless of the epegRNA used, all plants displayed the desired edit (Supplementary Fig. S7F). Taken together, these results show that, if editing rate variation between epegRNAs can be expected, the precise editing of any targetable locus is possible using a standard protoplast transfection experiment.

### Improving peg and epegRNA primer editing efficiency

**Fig. 4.**
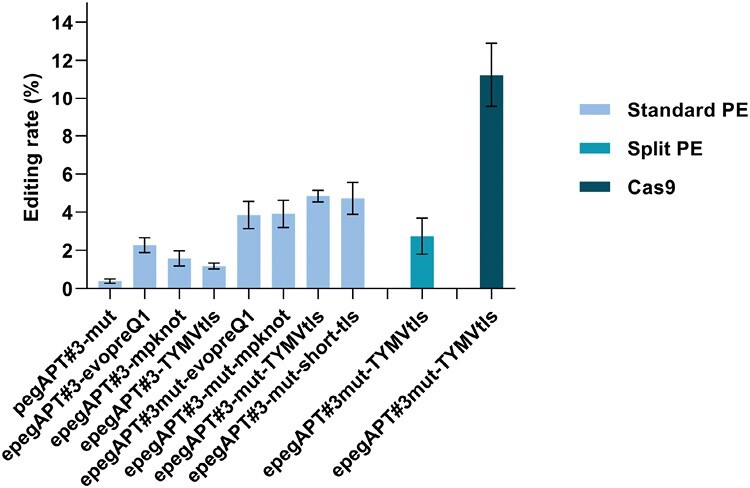
Prime editing (PE) efficiency of pegAPT#3-mut, epegAPT#3s, and epegAPT#3-mut using standard or split PE. With the pegRNA#3s, the PE rate is equal to the mutation rate as all the *apt* mutants displayed the expected edits. Data from three independent experiments. Cas9 nuclease is shown for comparison. Error bars=SE.

Inclusion of silent edit positions in addition to the target positions in the RT template has been shown to increase the efficiency of PE (X. Li *et al*., 2022b; Xu *et al.*, 2022). A possible explanation behind this phenomenon is that increasing the number of mutated bases leads to the saturation of the endogenous repair mechanism, favoring the insertion of the edited template versus the non-edited DNA sequence (Chen *et al.*, 2021). To evaluate this effect, we added two supplementary nucleotide changes to the existing pegAPT#3 to create pegAPT#3-mut (see Supplementary Table S1 for sequence information). Additionally, we created a –mut version for each of the three epegAPT#3s. After protoplast transfection and direct selection (Supplementary Fig. S5A), the PE rates using pegRNA#3-mut did not show any improvement over pegRNA#3, with an editing rate of 0.4% (Supplementary Table S4). In contrast, the editing rate obtained with the three epegAPT#3-muts were more than double compared with their epegAPT#3 counterparts (Fig. 4), suggesting a potential synergistic effect between the addition of mutations and the 3ʹ end extension. Notably, epegAPT#3-mut-TYMVtls displayed the highest PE rate of 4.85% in the present study, a value exceeding 40% of the mutation rate generated with a standard Cas9-targeted mutagenesis for the same guide (Fig. 4). The quality of the editing was not affected by the addition of two SNPs in pegAPT#3-mut as all the tested edited plants displayed the correct four nucleotide changes (Supplementary Table S4). The epegAPT#3-mut-TYMVtls yielded the highest editing rate but it is also the one with the longest 3ʹ extension, 86 nucleotides (Supplementary Table S2). We tried to shorten this sequence so it would contain only the last 41 nt of the TYMVtls (Supplementary Table S2), an extension named short-tls. The resulting epegAPT#3-mut-short-tls PE rate was nearly identical to that of epegAPT#3-mut-TYMVtls, and with the perfect editing pattern at the locus (Fig. 4; Supplementary Fig. S7D). It shows that the short-tls 3ʹ extension sequence provides protection similar to TYMVtls and could be used for further work.

### Split prime editing is functional in *P. patens*

Recent studies in mammalian cells indicate that it is possible to perform PE with the two domains of the prime editor, nCas9 and RT_MMLV_, as two individually expressed polypeptides, referred as split prime editing (sPE) with untethered RT (B. Liu *et al*., 2022). As this approach would greatly facilitate the testijng of nCas9 and RT variants in plants, we performed a protoplast transfection and direct selection procedure (Supplementary Fig. S5A) using pUbi-nCas9 and pUbi-RT_MMLV_ (Fig. 1A) as the split prime editor, and epegAPT#3-mut-TYMVtls as guide RNA. sPE was successful in our system, displaying an editing rate 50% that of standard PE (Fig. 4). We subsequently randomly selected 48 edited plants and sequenced the targeted locus: they all displayed the perfect desired pattern, indicating that the quality of the editing was not altered with this approach. As the editing rate observed remained high, these results open up the use of sPE as a base for high-throughput strategies to optimize the two components (nCas or RT) independently and to improve PE in plants.

### The native reverse transcriptase of the plant *Tnt1* retrotransposon is functional in a PE context

**Fig. 5.**
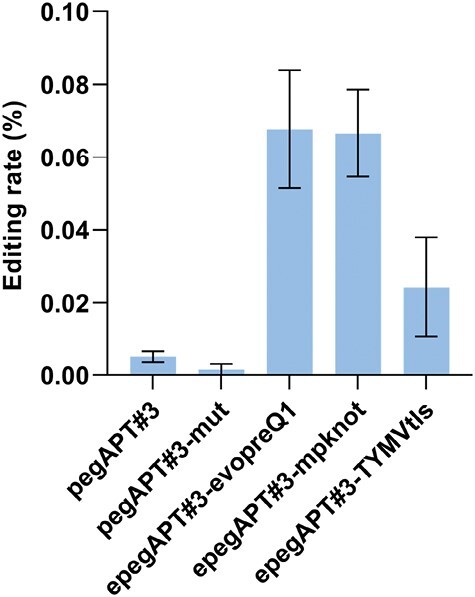
pUbi-PPETnt1 prime editing efficiency using alternative pegAPT#3s. With the pegRNA#3s, the prime editing rate is equal to the mutation rate as all the *apt* mutants displayed the expected edits. Data from three independent experiments. Error bars=SE.

As the only RTs used so far in the context of PE in plants are of either bacterial or viral origin, we wanted to see if the use of an RT of plant origin would be possible and potentially improve the PE rate in the green lineage. We chose the RT of the tobacco retrotransposon *Tnt1* (Grandbastien *et al.*, 1989), shown previously to be active in *P. patens* (Vives *et al.*, 2016). We assembled the vector pUbi-PPE_tnt1_ (Fig. 1A) based on the same architecture as pUbi-PPE_MMLV_ and we performed the protoplast transfection and direct selection procedure (Supplementary Fig. S5A) using pegAPT#3, pegAPT#3-mut, and the three epegAPT#3s. We obtained edited clones with all tested guide versions (Fig. 5), albeit at an editing rate that was 50–100 times below that observed with pUbi-PPE_MMLV_. These reduced rates were not surprising as the RT_MMLV_ used for PE has been optimized through mutagenesis (Anzalone *et al.*, 2019). Thus, RT_tnt1_ may be potentially an interesting alternative but will need further improvements if it is to be used on a routine basis. Use of epegRNA with pUbi-PPE_tnt1_ yielded a 10-fold increase in editing rate, which is the same range of increase as observed with pUbi-PPE_MMLV_ with the same epegRNA, a further indication of the importance of the pegRNA 3ʹ extension in the PE process.

## Discussion

Since the establishment of PE as a precise genome editing method in mammalian cells (Anzalone *et al.*, 2019) and the extension to diverse organisms such as plants (Lin *et al.*, 2020; Perroud *et al.*, 2022), it has been clear that a significant increase in editing rate would be necessary to make this approach a routine tool for fundamental and applied research (Molla *et al.*, 2021). Using the model plant *P. patens*, we present here several improvements that can be extended to other land plants. The search for the optimal promoter sequence for protein expression is a goal in any plant system, and *P. patens* is no exception. Of the early tested plant promoters in this species (Horstmann *et al.*, 2004), the rice Actin1 promoter was considered a strong promoter and has been used accordingly in different contexts, notably for our earlier PE demonstration (Perroud *et al.*, 2022). We observed in this study that the use of the maize ubiquitin promoter, another strong promoter in *P. patens* used recently with success to drive a Cas9 variant (Holá *et al.*, 2021), can increase the PE rate with standard pegRNA without affecting the quality of editing (Table 1). This increase indicates that protein accumulation can be a limiting factor in the PE approach and each plant will probably require such optimization. Finally, although it was not evaluated in the present study, the optimization of the promoter driving the guide RNA should not be ignored. For example, an engineered U6 promoter to drive the guide RNA in rice significantly increased the PE rate with all tested pegRNAs in transient assays (J. Li *et al*., 2022).

The strongest increase in PE rate was observed with the use of epegRNA (Nelson *et al.*, 2022), that contains the addition of a 3ʹ extension at the end of the pegRNA. All tested epegRNAs increased PE efficiency by factors between 4-fold and 150-fold (Figs 2, 4), surpassing what has been observed in rice, with a maximum 10-fold increase in stably transformed plants (J. Li *et al*., 2022; Zong *et al.*, 2022). Moreover, this PE rate increase did not affect the quality of the editing. Similarly to what has been observed with standard pegRNA (Perroud *et al.*, 2022), by-products, such as the generation of either partial or unwanted mutations at the targeted site, remained overall low in frequency and target dependent, a pattern already observed in rice (X. Li *et al*., 2022b; Xu *et al.*, 2022; Zong *et al.*, 2022). The data obtained with the epegAPT#3s are particularly encouraging: considering all the different epegAPT#3s variants tested in the course of the present study, we sequenced 546 different, independently edited plants at this targeted site, and all of them corresponded to the desired editing. This number of desired editing events indicates that PE can be perfectly predictive when the target sequence allows it. Further structural analysis of the target sequence APT#3 may reveal insights to optimize future target sequence design.

The successful use of the TYMVtls extension and its shortened version short-tls as a stabilizing 3ʹ pegRNA extension, which conferred similar PE rate increases to the established evopreQ1 and mpknot, adds a new option to the repertoire of pegRNA-stabilizing 3ʹ extension. Furthermore, this observation strengthens the idea that an mpknot RNA structure remains a good choice for RNA stability in the context of the guide RNA design compared with other RNA extensions such as G-PE (X. Li *et al*., 2022a). Finally, we note that the 3ʹ end of the pegRNA can be extended significantly as the size of the full TYMVtls, 84 nucleotides, did not show any PE rate reduction compared with the other 3ʹ extensions that are half this length.

The high level of PE conferred by the epegRNAs prompted us to try to edit a gene using indirect selection as commonly used for CRISPR/Cas editing in *P. patens* (Collonnier *et al.*, 2017; Mara *et al.*, 2019; Guyon-Debast *et al.*, 2021). This approach was successful using two different targets, the *PpAPT* gene with the epegAPT#3-TYMVtls and the *PpDEK*1 gene with the epegDEK1-TYMVtls (Fig. 3). In both cases, the editing-induced phenotypes were indistinguishable from their homologous recombination-inducible counterparts, for example resistance to 2-FA for the edited *PpAPT* and *dek*^*0*^ for the edited *PpDEK1.* The number of edited plants for the two epegRNAs differed greatly, as observed previously, but all tested plants were correctly edited. Together, this indicates that PE with indirect selection shows rates high enough to be used on novel targets for highly precise gene function analysis in *P. patens.*

The best PE frequency observed in this study reached 40% of CRISPR/Cas9-induced mutations, indicating that there is still room for improvement for PE. One avenue is the understanding of the cause(s) of the high variation of editing frequency from one epegRNA to another. One explanation possibly lies in the interaction between each guide and the nCas9. Although the tracRNA has been optimized for efficiency (Anzalone *et al.*, 2019), several authors have suggested improvement either in the PE framework or with regular Cas9. Notably, the modification of the central stem–loop of the tracRNA can be a way to increase editing efficiency either by increasing its size in the case of the so-called t-lock (Riesenberg *et al.*, 2022) or by adding an additional G–C at the base of its stem (X. Li *et al*., 2022b).

Split PE has recently emerged as an alternative configuration to the standard PE approach in mammalian cells by expressing the prime editor as two independent polypeptides (B. Liu *et al*., 2022; Zheng *et al.*, 2022; Feng *et al*., 2023; Grünewald *et al*., 2023). This approach allows PE to be performed with a virus-encoded prime editor as sPE diminishes the viral particle sequence size limitation. sPE will also facilitate greatly the rapid test for each prime editor functional unit. For the first time, we show in this study that sPE is functional in plants. Although we observed a 50% editing rate reduction compared with the standard PE, this editing rate remained very high and will permit the use of this approach for further optimization of the system.

The present study demonstrates that the PE rate can be improved through an incremental process. With the improved rate, we show that the coupled use of a strong promoter and epegRNA permits the design and execution of the editing of potentially any loci in the genome using a method of selection of transiently transfected cells. We have shown that the PE rate can be increased further in plants. For example, the modification of the RT-_MMLV_ by removing its subdomain responsible for its RNase H activity coupled with the addition of a viral nucleocapsid protein between nCas9 and the remainder of the RT_MMLV_ led to a modest but significant increase of the PE activity in rice and wheat (X. Li *et al*., 2022b; Zong *et al.*, 2022). The first successful use of native plant RT in the context of plant PE, albeit not a low editing rate, opens up the possibility of further improvement either through the optimization of the *Tnt1* RT or with the screening of the large array of RTs present in plant genomes. Moreover, the demonstration that sPE is functional in plants will help such screens for better prime editors.

## Supplementary data

The following supplementary data are available at *JXB* online.

Table S1. Sequences of pegRNAs used for *PpAPT* and *PpDEK1* prime editing.

Table S2. 3ʹ epegRNA extension sequences.

Table S3. Primers used in this study.

Table S4. Prime editing efficiency and quality of pegAPT#3 variants using fused and split PE systems in *P. patens*.

Fig. S1. Maps of the expression vectors used in this study.

Fig. S2. Tnt1 reverse transcriptase domain sequences from the Tnt1-94 retrotransposon element from tobacco (X13777).

Fig. S3. Prime editing strategy for the precise modifications of *PpAPT* and *PpDEK1* genes.

Fig. S4. Plasmid maps of pegRNA and epegRNA expression constructs.

Fig. S5. Plant selection procedures used after transient transfection of moss protoplasts.

Fig. S6. Prime editing quality using pUbi-PPE_MMLV_ and four APT-pegRNAs.

Fig. S7. Examples of edited plants using prime editing.

Fig. S8. Examples of sequenced predicted off-target loci of *apt* mutant plants using prime editing with epegAPT#2.

Fig. S9. Prime-edited *dek*^*0*^ is indistinguishable from the homologous recombination-generated *dek*^*0*^.

erad189_suppl_Supplementary_Tables_S1-S4_Figures_S1-S9

## Data Availability

The data and molecular tools that support the findings of this study are available from the corresponding author upon reasonable request. A detailed protocol of *P. patens* tissue production, tissue protoplasting, protoplast transfection, and plant selection is available at Protocols DOI: dx.doi.org/10.17504/protocols.io.4r3l27r5qg1y/v1.
