# Supplementary material for: Improved prime editing allows for routine predictable gene editing in Physcomitrium patens
Source: J Exp Bot. 2023 May 27;74(19):6176–87. doi: 10.1093/jxb/erad189 (PMC10575697; doi:10.1093/jxb/erad189)
Supplement: erad189_suppl_Supplementary_Tables_S1-S4_Figures_S1-S9 [file erad189_suppl_supplementary_tables_s1-s4_figures_s1-s9.pdf]

Supplementary Data

Table S1*:* Sequences of pegRNAs used for *PpAPT* and *PpDEK1 Prime Editing*

Table S2*:* 3' epegRNA extension sequences

Table S3*:* Primers used in this study

Table S4*: Prime Editing* efficiency and quality of pegAPT#3 variants using fused and split PE systems in *P. patens*.

Fig. S1*:* Maps of the expression vectors used in this study.

Fig. S2*:* Tnt-1 Reverse transcriptase domain sequences from Tnt1-94 retrotransposon element from tobacco (X13777).

Fig. S3*: Prime Editing* strategy for the precise modifications of *PpAPT* and *PpDEK1* genes.

Fig. S4*:* Plasmid maps model of pegRNA and epegRNA expression constructs.

Fig. S5*:* Plant selection procedures used after transient transfection of moss protoplasts.

Fig. S6*:* Examples of edited plants using *Prime Editing*.

Fig. S7*: Prime Editing* quality using pUbi-PPE_{MMLV} and four APT-pegRNAs.

Fig. S8*:* Examples of sequenced predicted off-targets locus of *apt* mutant plants using *Prime Editing* with epegAPT#2.

Fig. S9*: Prime edited* dek^0 is indistinguishable from the homologous recombination generated dek^0.

Table S1: Sequences of pegRNAs used for *PpAPT* and *PpDEK1 Prime Editing*

Name	Target sequence + PAM	RT template sequence	PBS sequence
**pegAPT#1**	ATGTCTTAGGCCCTGTGATTAGG	TACTAcACTCTTagCTAAT	CACAGGGCCTAAG
**pegAPT#2**	GTGAAGATGTCGGCCTCCAAGG	GTCGCCATCCTTGgtatG	AGGCCGACATCTTC
**pegAPT#3**	TGTCTTAGGCCCTGTGATTAGGG	TATACTCTTCtCaAA	TCACAGGGCCTAAG
**pegAPT#3-mut**	TGTCTTAGGCCCTGTGATTAGGG	TATACTCTTCttagA	TCACAGGGCCTAAG
**pegAPT#8**	GCAGCTCCAAGAGTGCCTCCGGTGG	GACTTAATaGCACCG	GAGGCACTCTTGGA
**pegDEK1**	AGGGCCACCTAGGAGATTGCTGG	CCTAGGAGATTct	TGGTTTTTGA

Table S2: 3' epegRNA extension sequences

Extension name	Spacer sequence	3' guide extension sequence (5'-3')
evopreQ1	tctctctc	TTGACGCGGTTCTATCTAGTTACGCGTTAAACCAACTAGAAA
mpknot	tctctctc	GGGTCAGGAGCCCCCCCCCTGAACCCAGGATAACCCTCAAAGTCGGGGGG CAACCC
TYMV-tls	tctctctc	GTTAGCTCGCCAGTTAGCGAGGTCTGTCCCCACACGACAGATAATCGGGTG CAACTCCCGCCCCTCTTCCGAGGGTCATCGGAACC
short-tls	tctctctc	CGGGTGCAACTCCCGCCCCTCTTCCGAGGGTCATCGGAACC

Table S3: Primers used in this study

Name	5'-> 3' sequence
PpAPT#5	ACAAGGTGGTGTCAACTTTCAAGG
PpAPT#14	AGATGTCGGCCTCCAAGGATG
PpAPT#60	ATGGTCAATGTGGCAGCAAG
PpAPT#61	CCTGTCAACCCTTACCTGGA
PpRad51#6	TGAGGAGGAAGTTCATCATGG
PpRad51#7	ACCGCCAATGGGTTTATGC
PpDEK1-Fwd	CGAACATGCCCTTCTAGAAAGAAG
PpDEK1-Rev	CATCATTATGATCTTCATCCTCATACCTG

Table S4: *Prime Editing* efficiency and quality of pegAPT#3 variants using fused and split PE systems in *P. patens*.

Name	pUbi-PE [a]			Split PE [a]		
	nb 2FA^R [b]	Mut. Freq. % [c]	PE % (n) [d]	nb 2FA^R [b]	Mut. Freq. % [c]	PE % (n) [d]
**pegAPT#3**	719	0,420 (±0,075)	100% (39)			
**pegAPT#3-mut**	155	0,390 (±0,127)	100% (37)			
**epegAPT#3-mut-evopreQ1**	1289	3,85 (±0,612)	100% (40)			
**epegAPT#3-mut-mpknot**	2354	3,92 (±0,619)	100% (42)			
**epegAPT#3-mut-TYMVtls**	2393	4,85 (±0,261)	100% (44)	581	2,74 (±0,817)	100% (44)
**epegAPT#3-mut-tls**	1782	4,73 (±0,725)	100% (41)			

[a] Data from 3 independent transfections.
[b] 2-FA^R stands for the total number of 2-FA resistant plants obtained during the three transfections.
[c] Mutation frequency (Mut. Freq.) is the % of *apt* mutants (2-FA^R) among the regenerated plants.
[d] *Prime Editing* efficiency (PE) is the % of plants with the expected edits among the *apt* mutants. n for number of sequenced plants.

A

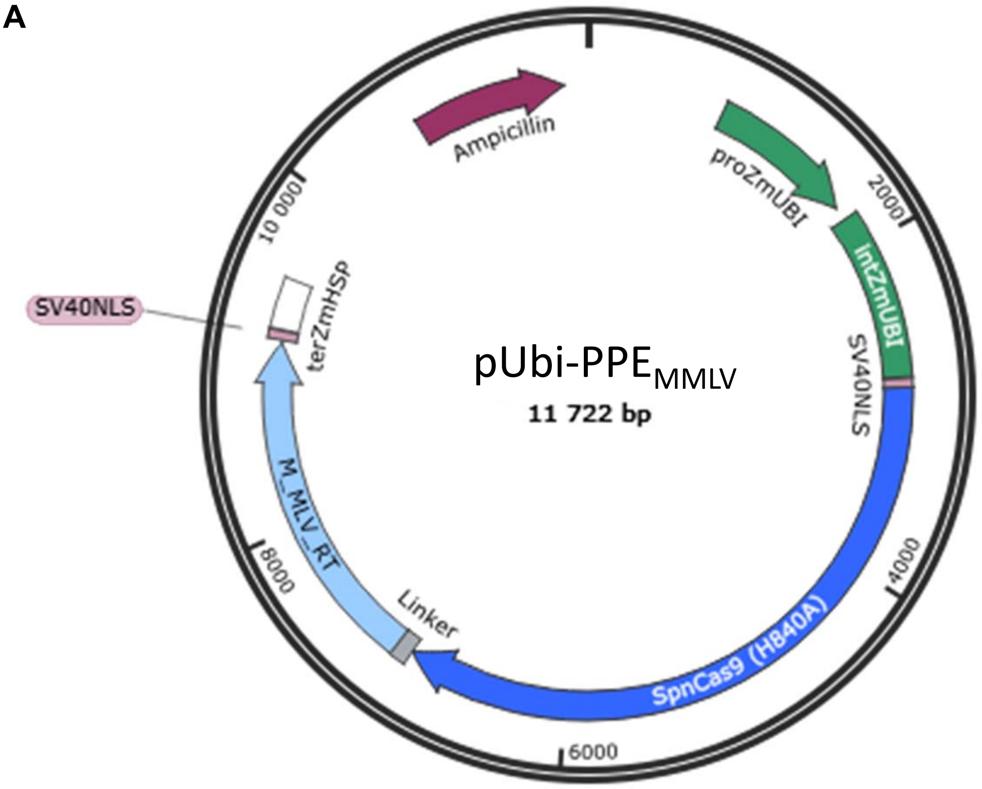

Ampicillin
proZmUBI
2000
IntZmUBI
SV40NLS
10 000
SV40NLS
terZmHSP
pUbi-PPE$_{MMLV}$
11 722 bp
M_MLV_RT
8000
Linker
SpnCas9 (H840A)
4000
6000

B

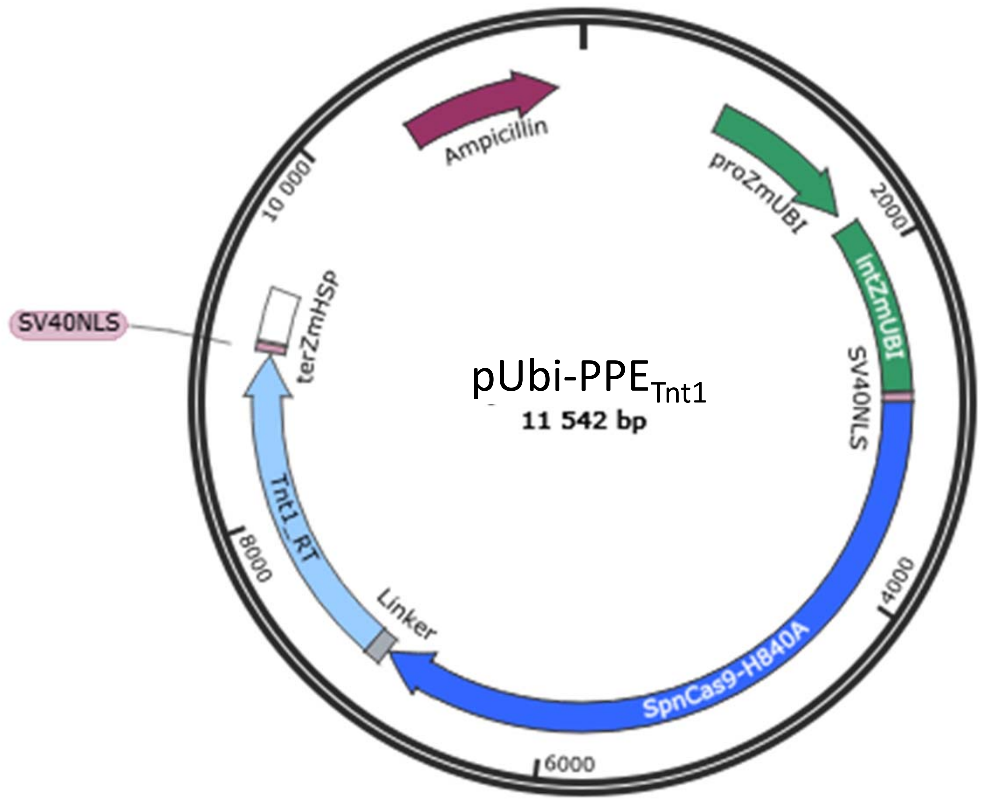

Ampicillin
proZmUBI
2000
IntZmUBI
SV40NLS
10 000
SV40NLS
terZmHSP
pUbi-PPE$_{Tnt1}$
11 542 bp
Tnt1_RT
8000
Linker
SpnCas9-H840A
4000
6000

C

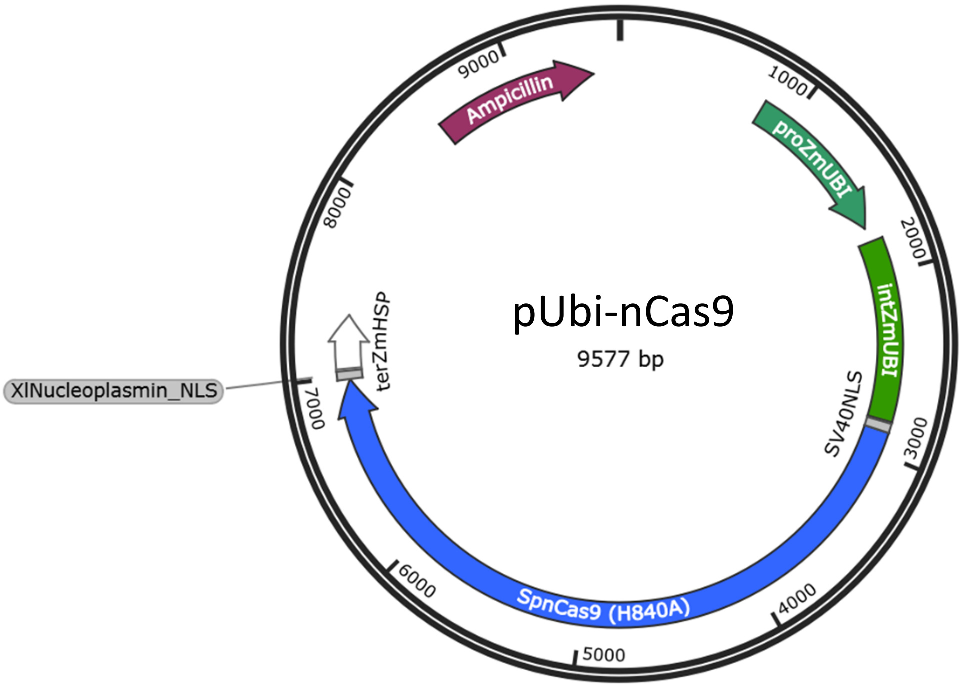
pUbi-nCas9
9577 bp
Ampicillin
proZmUBI
intZmUBI
SV40NLS
SpnCas9 (H840A)
terZmHSP
XlNucleoplasmin_NLS
1000
2000
3000
4000
5000
6000
7000
8000
9000

D

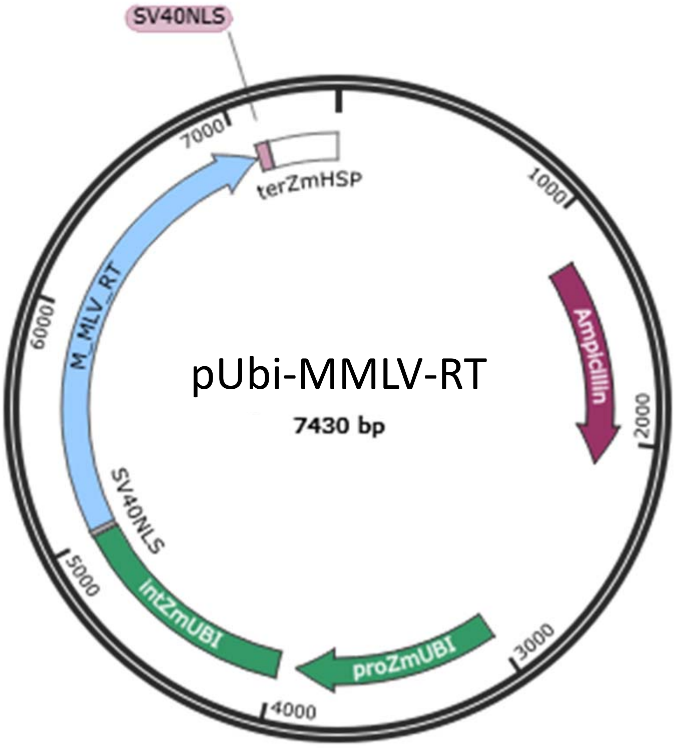
pUbi-MMLV-RT
7430 bp
SV40NLS
terZmHSP
M_MLV_RT
SV40NLS
intZmUBI
proZmUBI
Ampicillin
1000
2000
3000
4000
5000
6000
7000

E

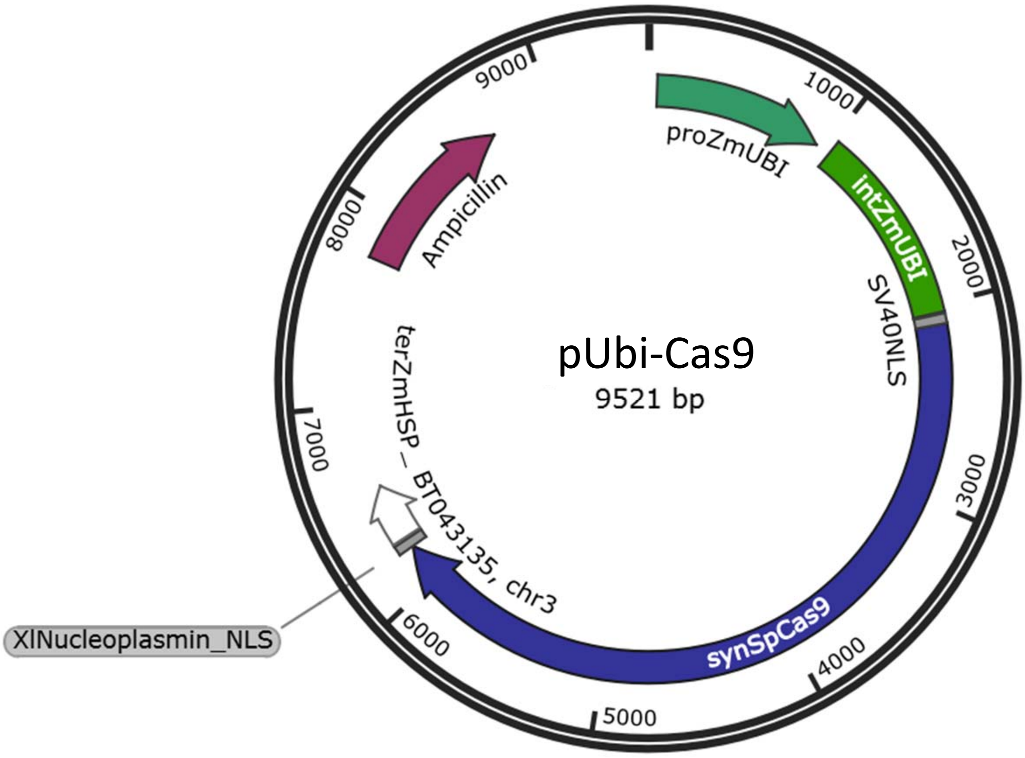

Fig. S1. Maps of the expression vectors used in this study.
(A) Plasmid map of pUbi-PPE$_{MMLV}$.(B) Plasmid map of pUbi-PPE$_{Tnt1}$. (C) Plasmid map of pUbi-nCas9. (D) Plasmid map of pUbi-MMLV-RT. (E) Plasmid map of pUbi-Cas9.

A

cccgcggacatgtccgagaaggtgaagaacggcatcatcccgaacttcgtcaccatcccctccac
gagcaacaaccccacctcggccgagtcgacgaccgacgaggtgagcgagcagggcgagcagccag
gcgaggtcatcgagcagggcgagcagctggacgagggcgtggaggaggtggagcacccgacccag
ggcgaggagcagcaccagccactgaggaggtcggagcgcccgagggtggagtccaggaggtaccc
cagcaccgagtatgtgctcatctcggacgacagggagccggagtccctgaaggaggtgctcagcc
acccggagaagaaccagctcatgaaggcgatgcaggaggagatggagtccctgcagaagaacggc
acctacaagctggtcgagctcccgaagggcaagaggcccctcaagtgcaagtgggtgttcaagct
gaagaaggacggcgactgcaagctcgtccgctacaaggccaggctggtggtcaagggcttcgagc
agaagaagggcatcgacttcgacgagatcttctcccccgtggtcaagatgaccagcatcaggacc
atcctgtcgctggcggccagcctggacctcgaggtggagcagctggacgtcaagaccgccttcct
gcacggcgacctcgaggaggagatctacatggagcagcccgagggcttcgaggtggccggcaaga
agcacatggtctgcaagctgaacaagtcgctctacggcctcaagcaggccccgaggcagtggtac
atgaagttcgactccttcatgaagtcccagacctacctcaagacgtactccgacccgtgcgtgta
cttcaagaggttcagcgagaacaacttcatcatcctcctgctctacgtggacgacatgctcatcg
tcggcaaggacaagggcctgatcgcgaagctcaagggcgacctgtccaagagcttcgacatgaag
gacctcggcccggctcagcagatcctgggcatgaagatcgtgcgcgagaggacctccaggaagct
gtggctcagccaggagaagtacatcgagcgcgtgctcgagaggttcaacatgaagaacgccaagc
cggtgtccacgcccctggctggccacctgaagctcagcaagaagatgtgccccaccacggtcgag
gagaagggcaacatggctaaggtgccctactcctcggccgtgggctccctgatgtacgctatggt
gtgcacccgccccgacatcgctcacgccgtgggcgtggtcagcaggttcctcgagaacccgggca
aggagcactgggaggcggtgaagtggatcctcaggtacctccggggcaccaccggcgactgcctc
tgcttcggtggctccgacccgatcctgaagggctacaccgacgcggacatggccggcgacatcga
caaccgcaagtccagcaccggctacctgttcacgttctccggcggcgccatctcctggcagagca
agctccagaagtgcgtggctctgagcacgaccgaggccgagtacatcgcggccacggagacgggc
aaggagatgatctggctcaagcgcttcctgcaggagctgggcctccaccagaaggagtatgtggt
ctactgcgactcccagagcgcgatcgacctgtccaagaacagcatgtaccacgccaggaccaagc
acatcgacgtgcgctaccactggatcagggagatggtggacgacgagtccctgaaggtcctcaag
atcagcaccaacgagaaccccgccgacatgctcacgaaggtggtcccgaggaacaagttcgagct
ctgcaaggagctggtcggcatgcacagcaac

B

AADMSEKVKNGIIPNFVTIPSTSNNPTSAESTTDEVSEQGEQPGEVIEQGEQLDEGVEEVEHPTQ
GEEQHQPLRRSERPRVESRRYPSTEYVLISDDREPESLKEVLSHPEKNQLMKAMQEEMESLQKNG
TYKLVELPKGKRPLKCKWVFKLKKDGDCKLVRYKARLVVKGFEQKKGIDFDEIFSPVVKMTSIRT
ILSLAASLDLEVEQLDVKTAFLHGDLEEEIYMEQPEGFEVAGKKHMVCKLNKSLYGLKQAPRQWY
MKFDSFMKSQTYLKTYSDPCVYFKRFSENNFIILLLYVDDMLIVGKDKGLIAKLKGDLSKSFDMK
DLGPAQQILGMKIVRERTSRKLWLSQEKYIERVLERFNMKNAKPVSTPLAGHLKLSKKMCPTTVE
EKGNMAKVPYSSAVGSLMYAMVCTRPDIAHAVGVVSRFLENPGKEHWEAVKWILRYLRGTTGDCL
CFGGSDPILKGYTDADMAGDIDNRKSSTGYLFTFSGGAISWQSKLQKCVALSTTEAEYIAATETG
KEMIWLKRFLQELGLHQKEYVVYCDSQSAIDLSKNSMYHARTKHIDVRYHWIREMVDDESLKVLK
ISTNENPADMLTKVVPRNKFELCKELVGMHSN

Fig. S2. Tnt-1 Reverse transcriptase domain sequences from Tnt1-94 retrotransposon element from tobacco (X13777).
(A) Nucleotide sequence (5'-3'). (B) Protein sequence.

A

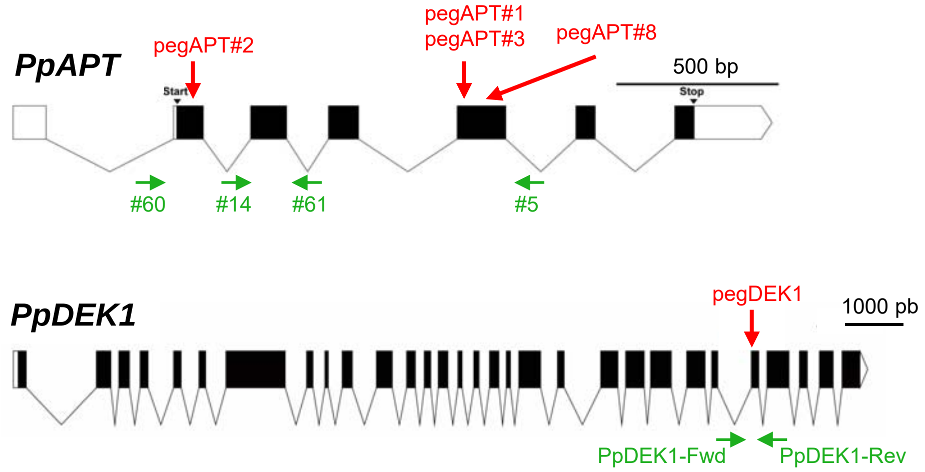

B

Name	Expected edit				RT template length	PBS length
	type	position	nature	Codon change		
**pegAPT#1**	3 snp	6	G-to-C	stop	18	13
		7	G-to-T			
		14	A-to-G	$Y^{103}C$		
**pegAPT#2**	ins4	2-5	ATAC	stop	18	14
**pegAPT#3**	2 snp	3	A-to-T	stop	15	14
		5	G-to-A			
**pegAPT#3-mut**	4 snp	2	T-to-C	-	15	14
		3	A-to-T	stop		
		4	G-to-A			
		5	G-to-A			
**pegAPT#8**	del1	5	- C	stop	16	14
	snp	8	T-to-A			
**pegDEK1**	2 snp	12	G-to-C	$C^{1782}S$	13	10
		13	C-to-T			

Fig. S3. *Prime Editing* strategy for the precise modifications of *PpAPT* and *PpDEK1* genes.
(A) Structure of the *PpAPT* and *PpDEK1* genes and pegRNAs positions. Boxes correspond to exon, line to intron. Black boxes define the gene open reading frames. The pegRNAs positions are indicated in red and primers used for PCR and sequencing in green. (B) pegRNAs expected edit information at the two tested loci, *PpAPT* and *PpDEK1*. The type and nature of mutations, position on RT product, length of RT template and PBS (position relative to the SSB site) provided for each pegRNA remain identical for their epegRNA counterpart. In *PpAPT*, all but pegAPT#2 generate a stop codon at the edit position. For the pegAPT#2, the 4bp-insertion generates a frameshift that creates a downstream stop codon at the amino acid position 24 in the first exon.

A

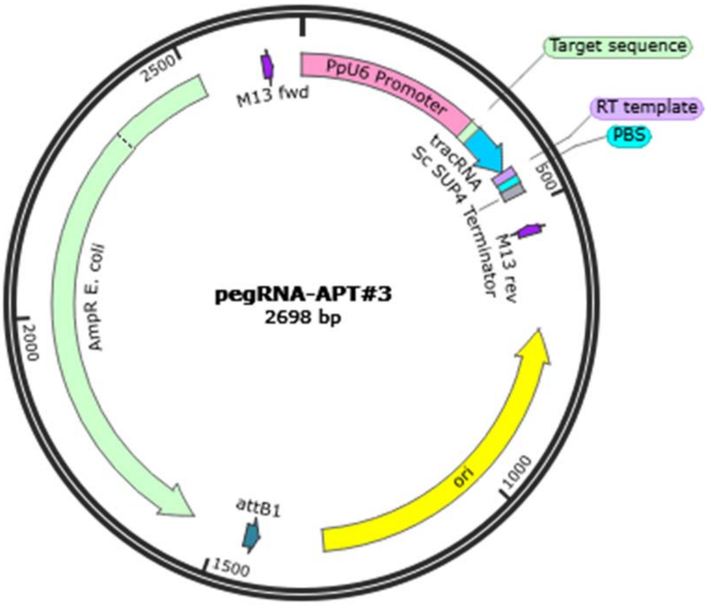

B

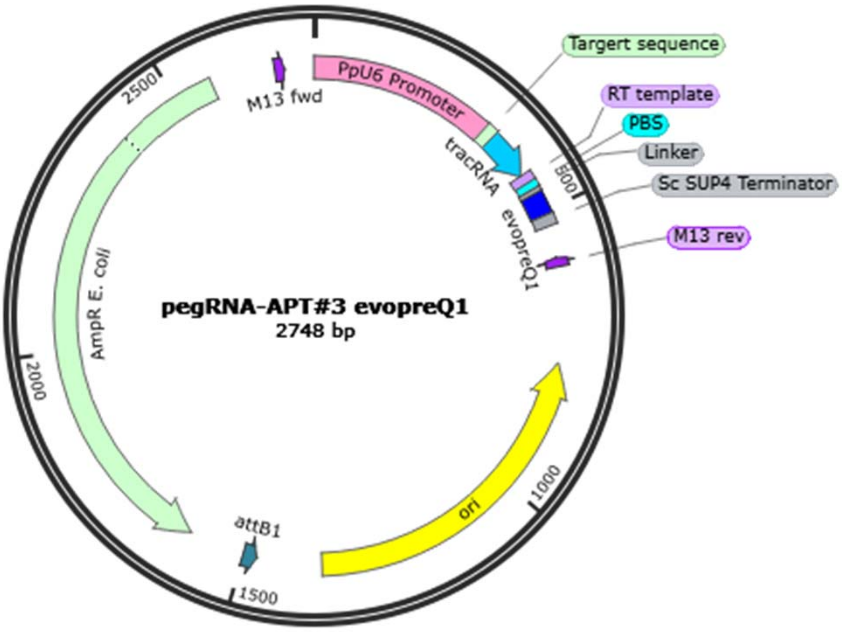

Fig. S4. Plasmid maps model for pegRNA and epegRNA expression constructs. (A) Plasmid map of pegAPT#3. (B) Plasmid map of epegAPT#3-evopreQ1.

A

B

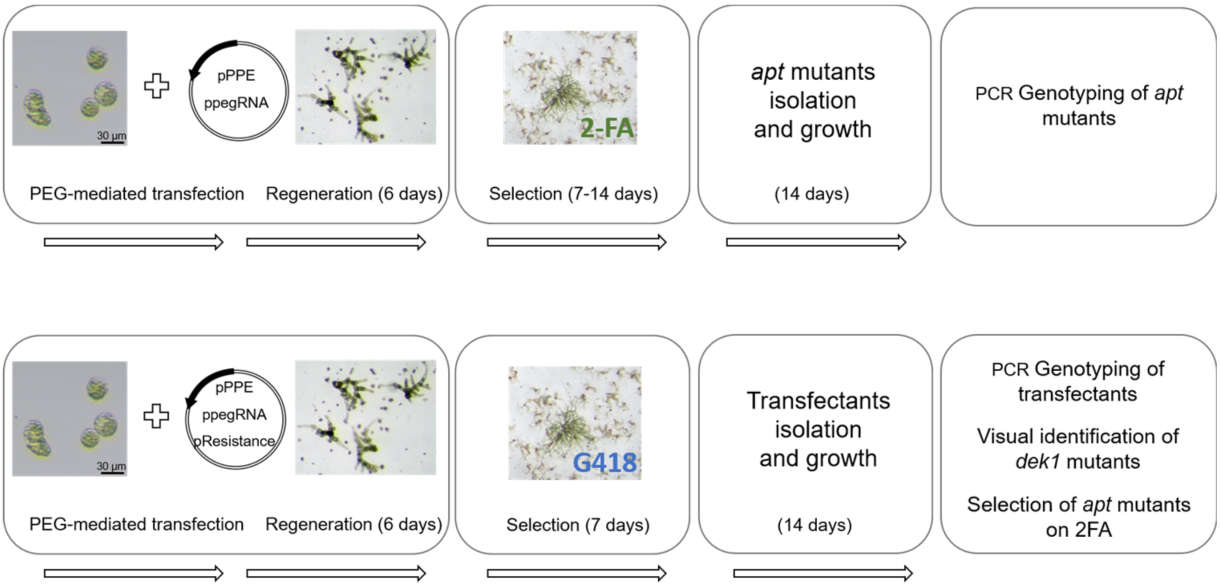

Fig. S5. Plant selection procedures used after transient transfection of moss protoplasts.
(A) Direct selection of *apt* mutants using 2-FA resistance conferred by *PpAPT* loss of function. (B) Transient selection of transfected plants using transient G418 resistance and identification of edited plants (*DEK1* or *APT* genes) in this population. Pictures of this figure are illustrating the different steps of the procedures and are not the actual samples/plants used in the study.

A

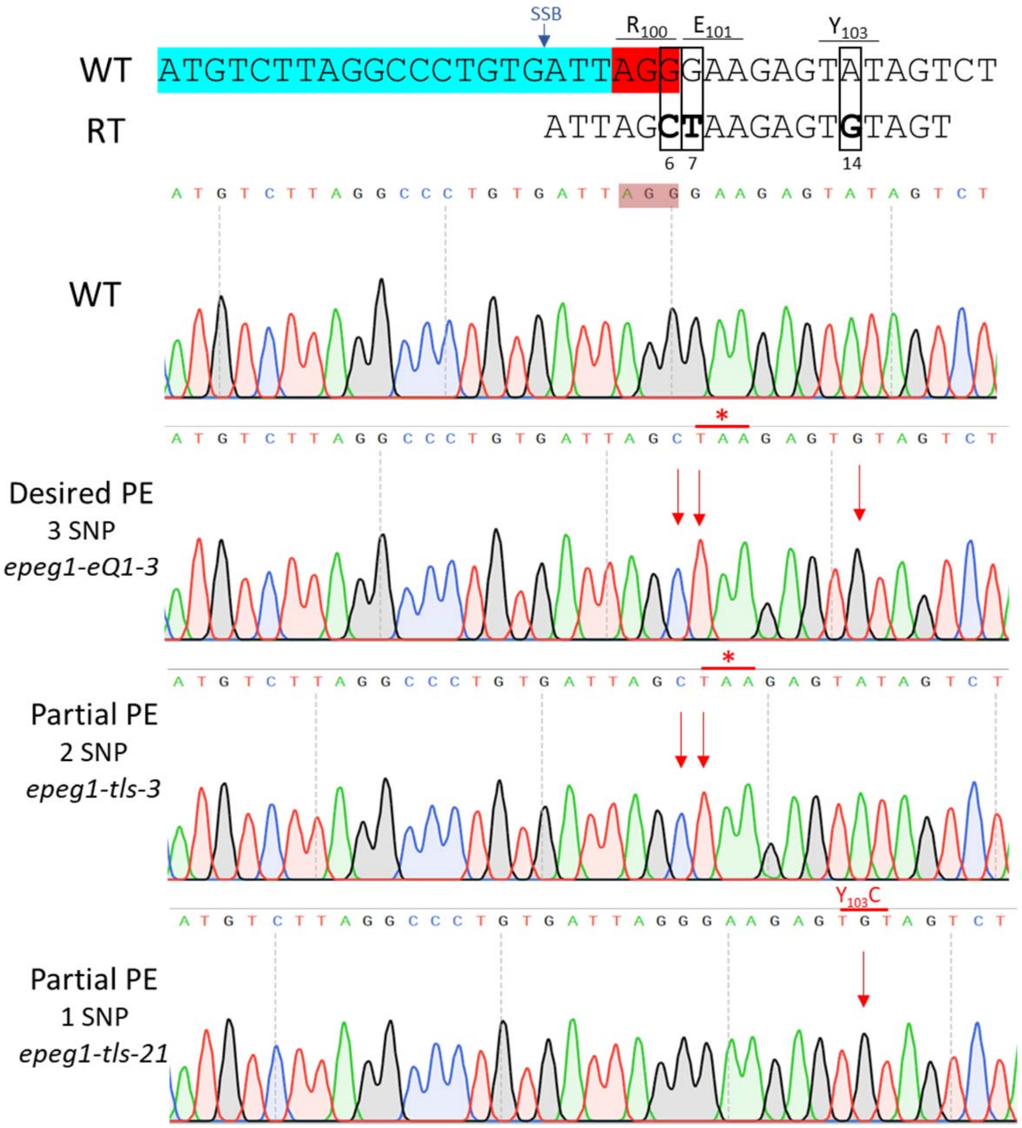

SSB
R100
E101
Y103
WT
ATGTCTTAGGCCCTGTGATTAGGGAAGAGTATAGTCT
RT
ATTAGCTAAGAGTGTAGT
6
7
14
WT
Desired PE
3 SNP
epeg1-eQ1-3
*
Partial PE
2 SNP
epeg1-tls-3
*
Partial PE
1 SNP
epeg1-tls-21
Y103C

B

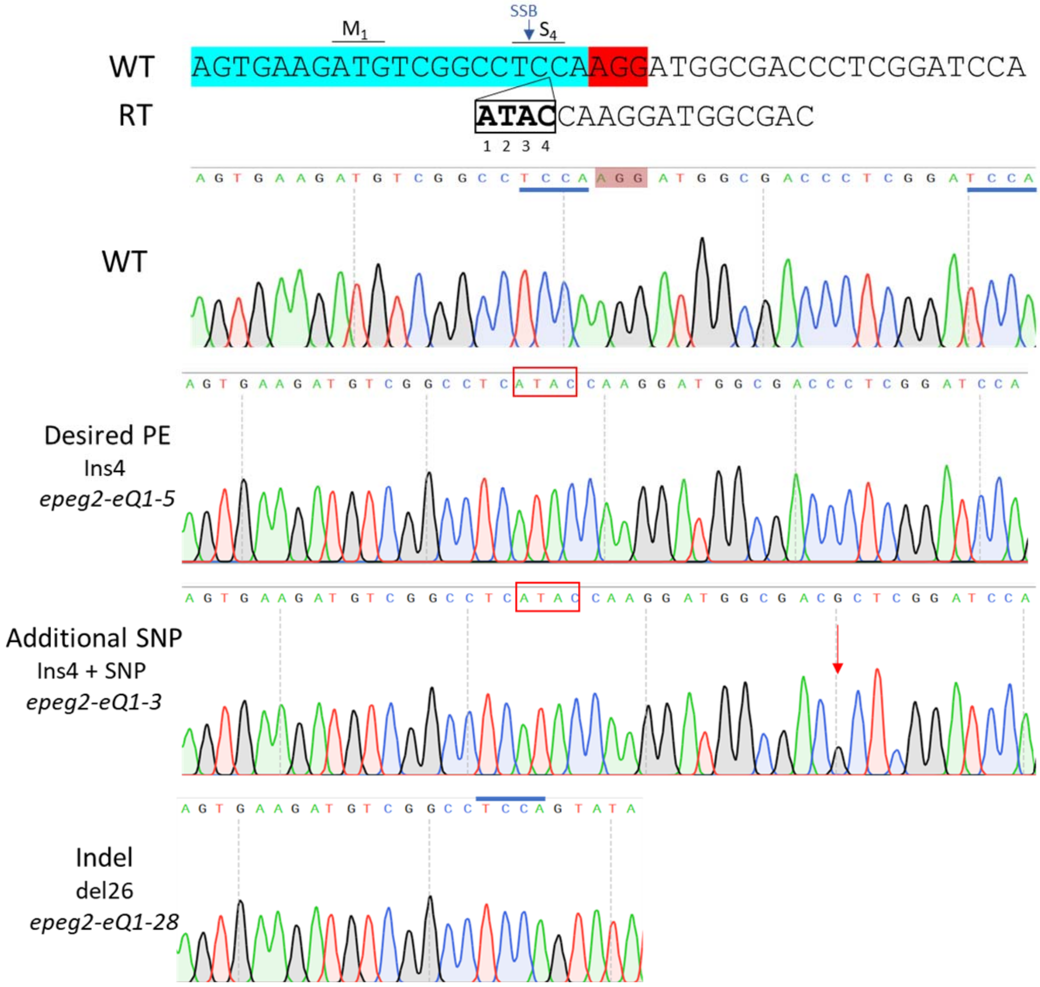
SSB
M1
S4
WT
AGTGAAGATGTCGGCCTCCAAGGATGGCGACCCTCGGATCCA
RT
ATACCAAGGATGGCGAC
1 2 3 4
AGTGAAGATGTCGGCCTCCAAGGATGGCGACCCTCGGATCCA
WT
AGTGAAGATGTCGGCCTCATACCAAGGATGGCGACCCTCGGATCCA
Desired PE
Ins4
epeg2-eQ1-5
AGTGAAGATGTCGGCCTCATACCAAGGATGGCGACGCTCGGATCCA
Additional SNP
Ins4 + SNP
epeg2-eQ1-3
AGTGAAGATGTCGGCCTCCAGTATA
Indel
del26
epeg2-eQ1-28

C

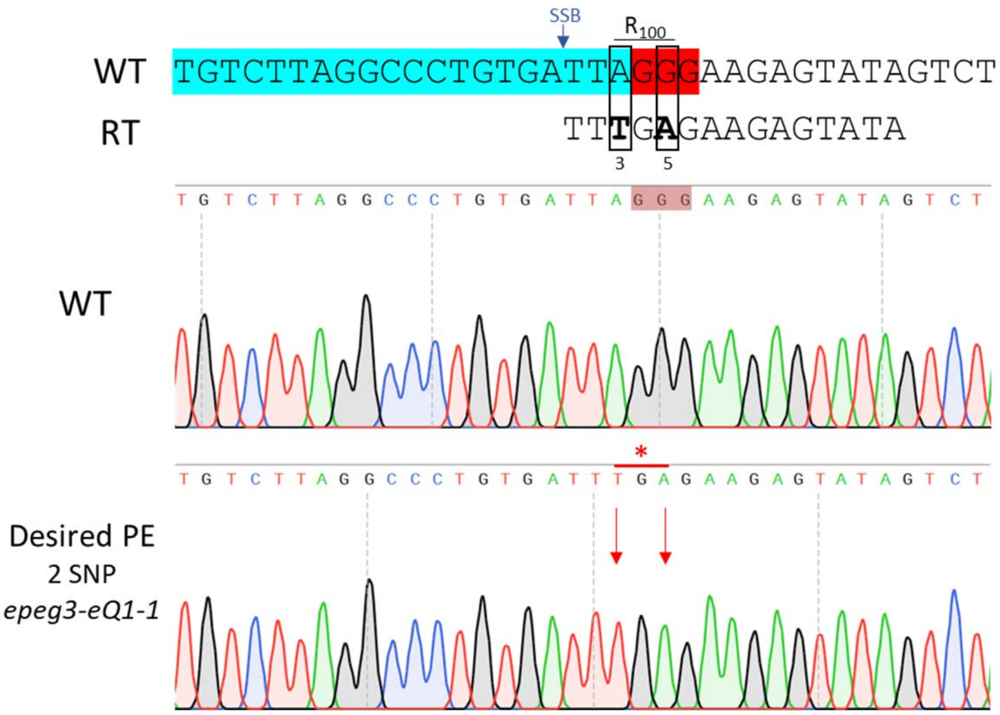
SSB
R100
WT TGTCTTAGGCCCTGTGATTAGGGAAGAGTATAGTCT
RT TTTGAGAAGAGTATA
3
5
TGTCTTAGGCCCTGTGATTAGGGAAGAGTATAGTCT
WT
*
TGTCTTAGGCCCTGTGATTTGAGAAGAGTATAGTCT
Desired PE
2 SNP
epeg3-eQ1-1

D

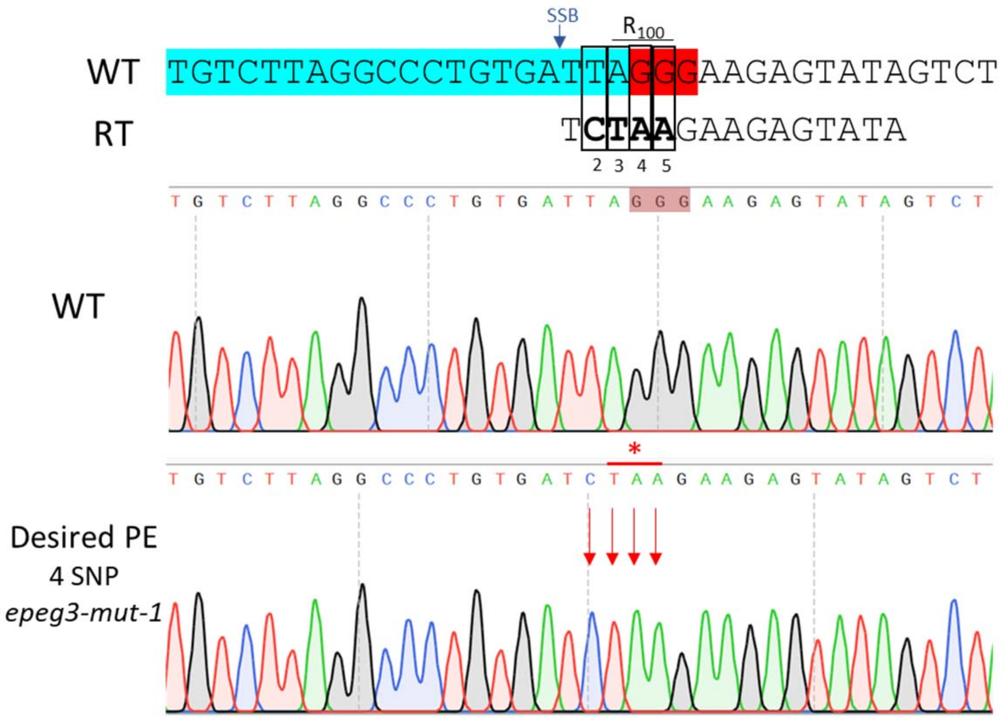
SSB
R100
WT TGTCTTAGGCCCTGTGATTAGGGAAGAGTATAGTCT
RT TCTAAGAAGAGTATA
2 3 4 5
TGTCTTAGGCCCTGTGATTAGGGAAGAGTATAGTCT
WT
*
TGTCTTAGGCCCTGTGATCTAAGAAGAGTATAGTCT
Desired PE
4 SNP
epeg3-mut-1

E

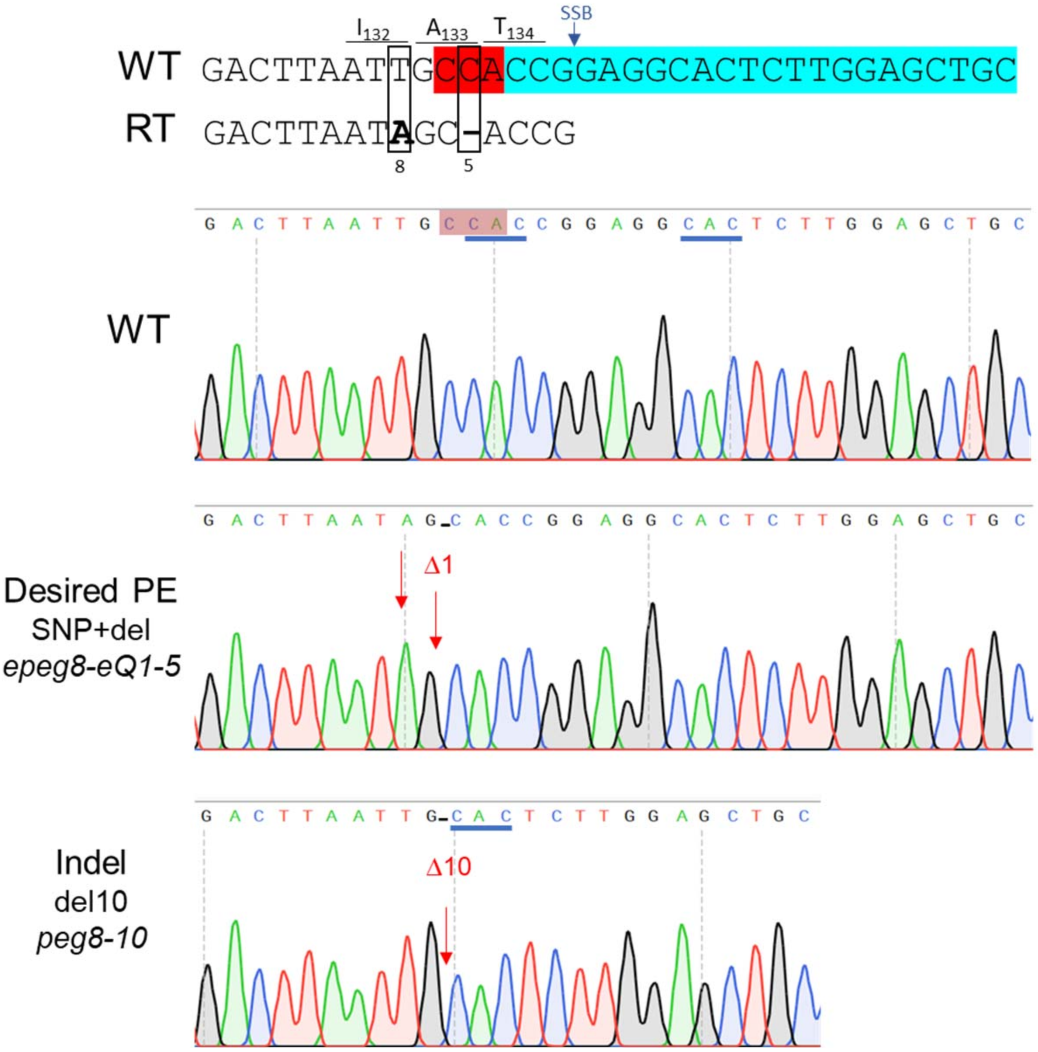
SSB
I_{132} A_{133} T_{134}
WT GACTTAATTGCCACCGGAGGCACTCTTGGAGCTGC
RT GACTTAATAGC-ACCG
8
5
WT
Desired PE
SNP+del
epeg8-eQ1-5
Δ1
Indel
del10
peg8-10
Δ10

F

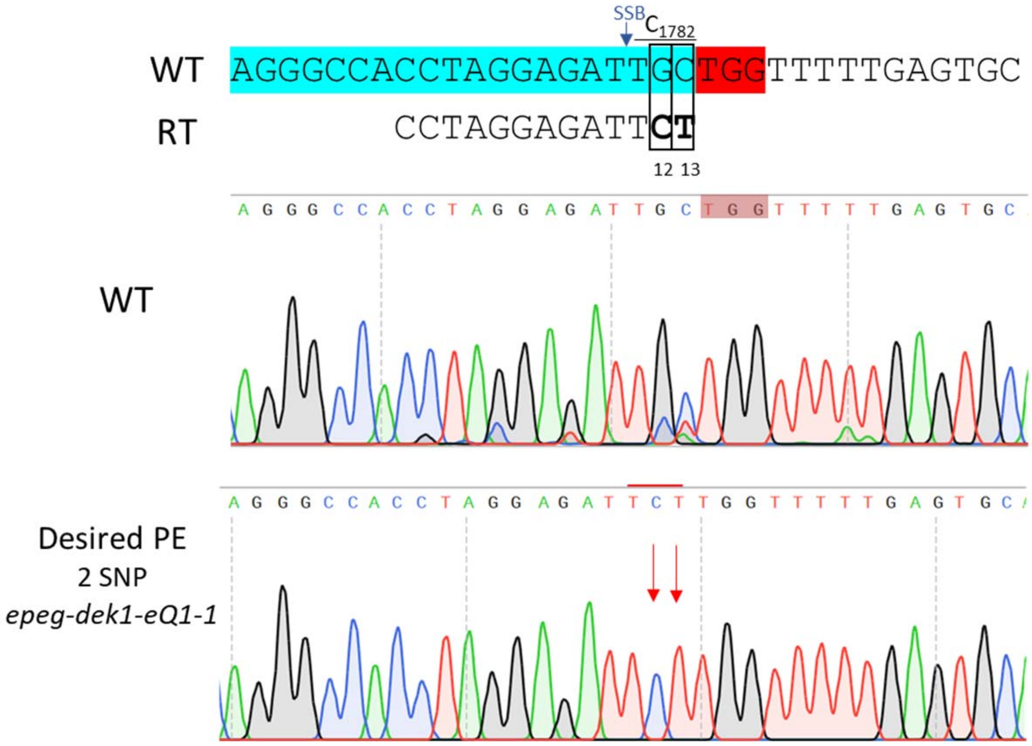

Fig. S6. Examples of edited plants using *Prime Editing*.

(A-F) : Schematic representation of the the wild-type genomic sequence (WT) and expected RT product (RT) using each pegRNA. (A) epegAPT#1. (B) epegAPT#2 (C) epegAPT#3. (D) epegAPT#3-mut. (E) epegAPT#8. (F) epegDEK1. Target sequence is highlighted in blue and PAM in red, blue arrow represents SSB site and relative position of mutations are precised below expected RT product sequence. A sequencing chromatogram of an edited plant using each pegRNA is shown below WT sequence, editions are indicated by red arrows or boxes.

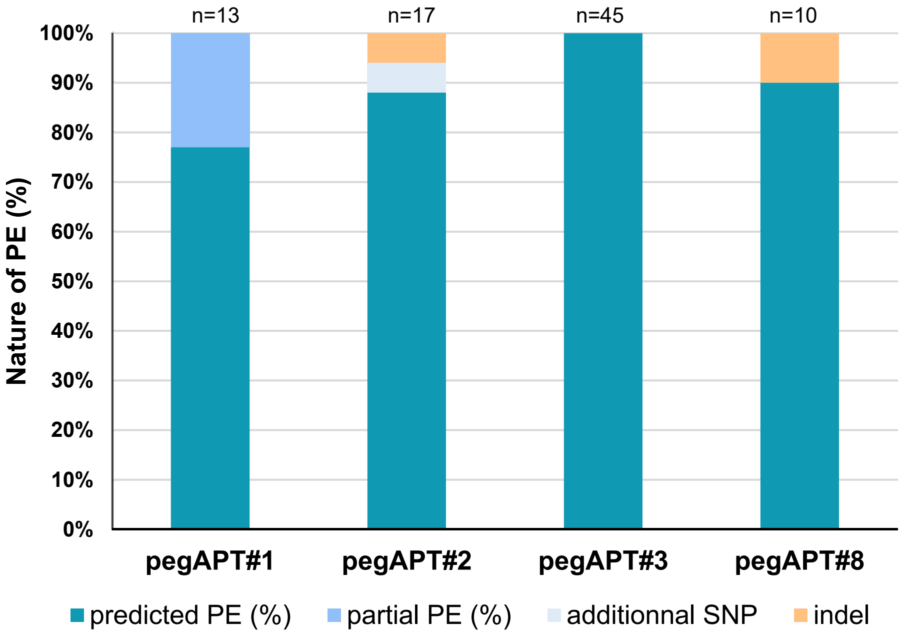

Fig. S7. *Prime Editing* quality using pUbi-PPE$_{MMLV}$ and four APT-pegRNAs.
Nature of PE for each pegRNA was assessed by PCR sequencing (Primers in Table S3). n: number of edited plants that were sequenced.

A peg2-OT1 (Chr03_16263830)

A**a**T**a**AAGATGTCGGC**a**TC**a**AAGG

WT

epeg2-5

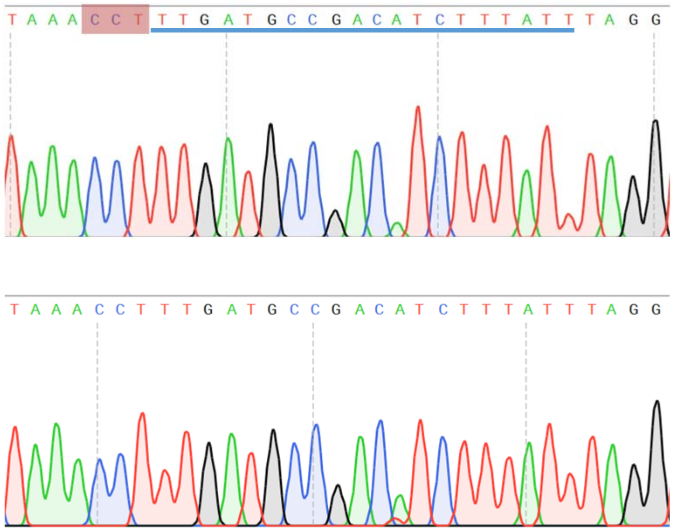

B peg2-OT2 (Chr01_24568979)

AGTG**g**AGA**gt**TCGGCC**a**CCACGG

WT

epeg2-5

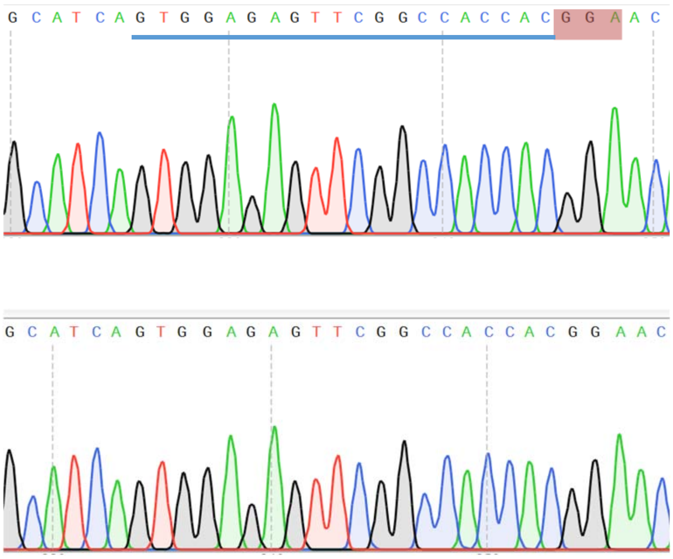

C peg2-OT3 (Chr02_10287524)

gGTGAA**a**ATG**g**C**t**GCCTCCACGG

WT

epeg2-5

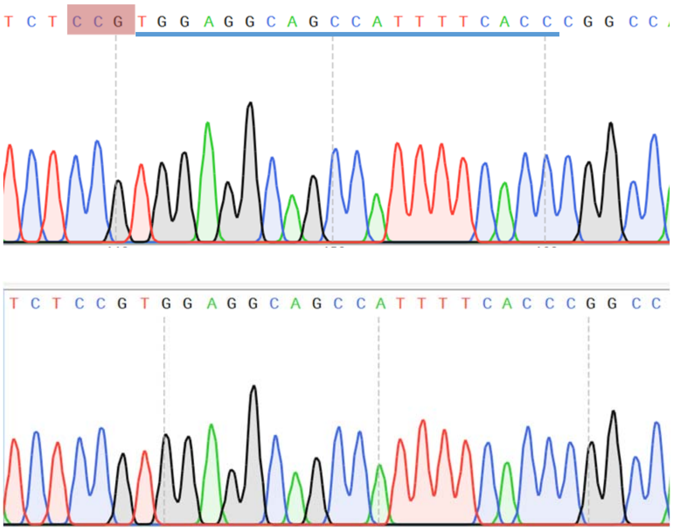

D peg2-OT4 (Chr15_8685405)

AGTGAAGA**g**GT**gac**CCTCCAGGG

WT

epeg2-5

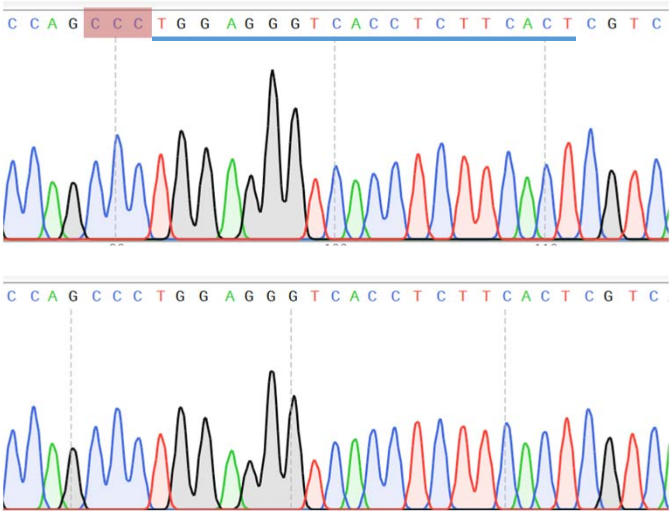

E

peg2-OT6 (Chr01_15854114)

A**t**TGAAGATGT**g**G**c**CC**a**CCACGG

WT

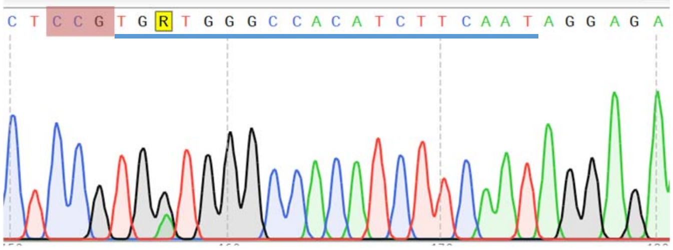

epeg2-5

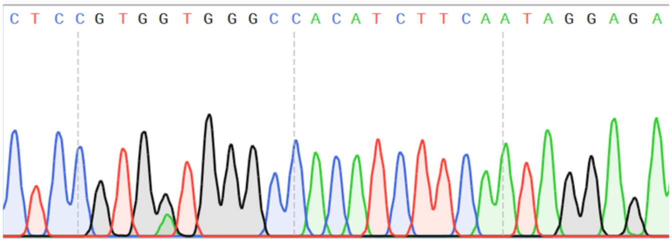

F

peg2-OT8 (Chr07_17136251)

AGTGA**t**GATG**g**C**t**GCCT**a**CATGG

WT

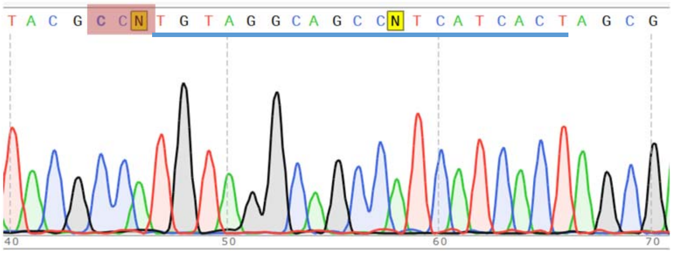

epeg2-5

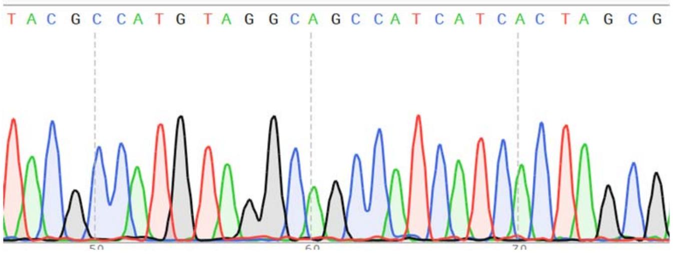

G peg2-OT9 (Chr10_12172644)

AGTGAAGAT**t**TCGGCCTCC**t**GGA

WT

epeg2-5

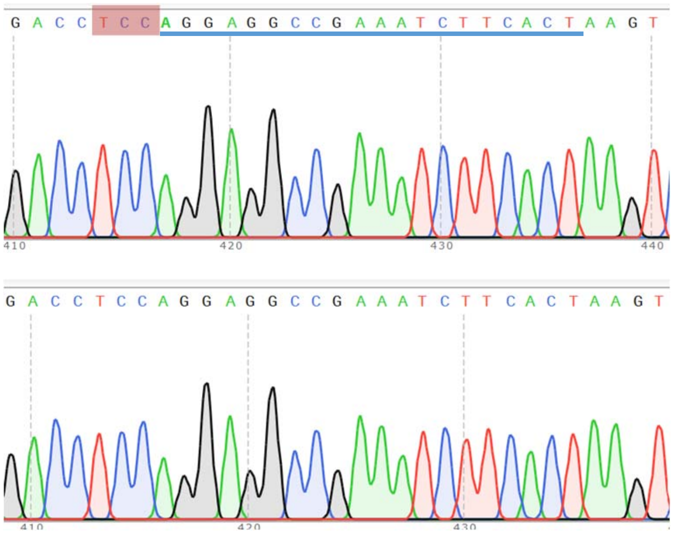

Fig. S8. Examples of sequenced predicted off-targets locus of *apt* mutant plants using *Prime Editing* with epegAPT#2.
(A-G) : Schematic representation of the the wild-type genomic sequence (WT) and one *apt* mutant plant for each off-target locus, as previously defined (Perroud *et al.* 2022). (A) peg2-OT1. (*B*) peg2-OT2. (C) peg2-OT3. (D) peg2-OT4. (E) peg2-OT6. (F) peg2-OT8. (G) peg2-OT9. Target sequence is highlighted in blue and PAM in red. A sequencing chromatogram of an *apt* mutant plant using epegRNA#2 is shown below WT sequence.

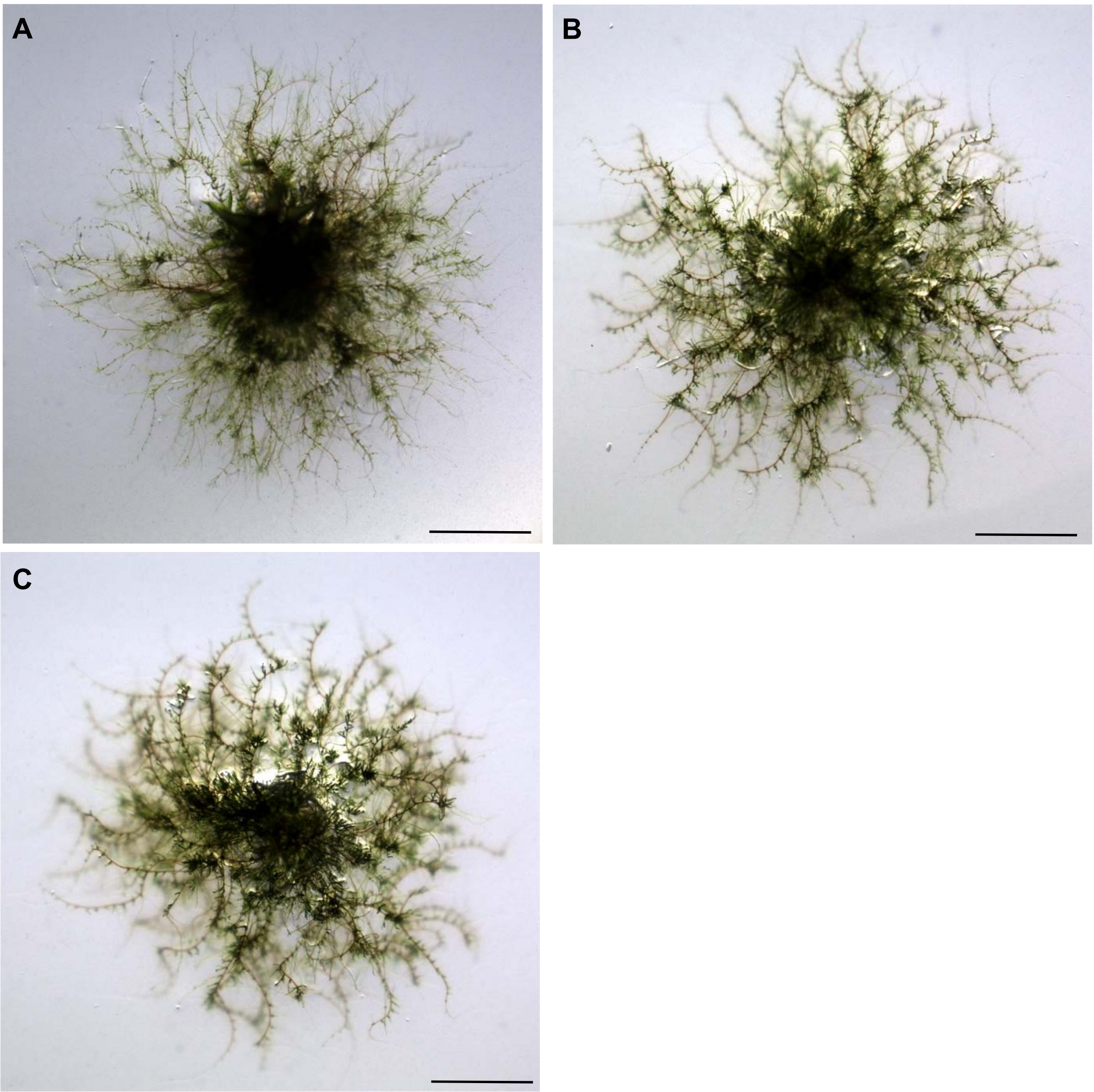

Fig. S9. *Prime edited dek^0* is indistinguishable from the homologous recombination generated *dek^0*. Plant grown from spot inoculum for 14 days on $PPN0_3$ medium. (A) Wild-type plant. (B) *dek^0* plant obtained by gene targeting. (C) *dek^0* plant obtained by *Prime Editing*. Bar: 2 mm.
